# Effects of Valproic Acid and Dexamethasone Administration on Early Bio-Markers and Gene Expression Profile in Acute Kidney Ischemia-Reperfusion Injury in the Rat

**DOI:** 10.1371/journal.pone.0126622

**Published:** 2015-05-13

**Authors:** Ryan W. Speir, Jonathan D. Stallings, Jared M. Andrews, Mary S. Gelnett, Timothy C. Brand, Shashikumar K. Salgar

**Affiliations:** 1 Department of Surgery, Madigan Army Medical Center, Tacoma, Fort Lewis, Washington, United States of America; 2 Environmental Health Program, US Army Center for Environmental Health Research, Fort Detrick, Maryland, United States of America; 3 Department of Pathology, Madigan Army Medical Center, Tacoma, Fort Lewis, Washington, United States of America; 4 Department of Clinical Investigation, Madigan Army Medical Center, Tacoma, Fort Lewis, Washington, United States of America; Universidade de Sao Paulo, BRAZIL

## Abstract

Renal ischemia-reperfusion (IR) causes acute kidney injury (AKI) with high mortality and morbidity. The objective of this investigation was to ameliorate kidney IR injury and identify novel biomarkers for kidney injury and repair. Under general anesthesia, left renal ischemia was induced in Wister rats by occluding renal artery for 45 minutes, followed by reperfusion and right nephrectomy. Thirty minutes prior to ischemia, rats (n = 8/group) received Valproic Acid (150 mg/kg; VPA), Dexamethasone (3 mg/kg; Dex) or Vehicle (saline) intraperitoneally. Animals were sacrificed at 3, 24 or 120 h post-IR. Plasma creatinine (mg/dL) at 24 h was reduced (P<0.05) in VPA (2.7±1.8) and Dex (2.3±1.2) compared to Vehicle (3.8±0.5) group. At 3 h, urine albumin (mg/mL) was higher in Vehicle (1.47±0.10), VPA (0.84±0.62) and Dex (1.04±0.73) compared to naïve (uninjured/untreated control) (0.14±0.26) group. At 24 h post-IR urine lipocalin-2 (μg/mL) was higher (P<0.05) in VPA, Dex and Vehicle groups (9.61–11.36) compared to naïve group (0.67±0.29); also, kidney injury molecule-1 (KIM-1; ng/mL) was higher (P<0.05) in VPA, Dex and Vehicle groups (13.7–18.7) compared to naïve group (1.7±1.9). Histopathology demonstrated reduced (P<0.05) ischemic injury in the renal cortex in VPA (Grade 1.6±1.5) compared to Vehicle (Grade 2.9±1.1). Inflammatory cytokines IL1β and IL6 were downregulated and anti-apoptotic molecule BCL2 was upregulated in VPA group. Furthermore, kidney DNA microarray demonstrated reduced injury, stress, and apoptosis related gene expression in the VPA administered rats. VPA appears to ameliorate kidney IR injury via reduced inflammatory cytokine, apoptosis/stress related gene expression, and improved regeneration. KIM-1, lipocalin-2 and albumin appear to be promising early urine biomarkers for the diagnosis of AKI.

## Introduction

The kidney is exquisitely sensitive to variations in blood flow [1]. Reduced or complete stoppage of blood flow to the kidney during perioperative conditions to prevent bleeding can lead to kidney damage and dysfunction. Significant research has been performed regarding different pharmacologic and hormonal agents that may be renoprotective in the setting of renal ischemia-reperfusion (IR). Renal IR injury is a complex syndrome which involves renal vasoconstriction, tubular damage, and glomerular injury. The reperfusion itself can often be as toxic to renal tubular cells as the ischemic insult. The renal injury is related to reactive oxygen radical production, loss of ATP, alterations in membrane lipids and phospholipase activation [2]. Currently, perioperative renal dysfunction prevention in patients with underlying chronic kidney disease is not well understood. Studies of perioperative renal dysfunction are complicated by the lack of a single endpoint scientifically or clinically proven to define renal dysfunction [3]. However, outcome measures commonly used in the evaluation of normal physiology and renal function include serum/plasma creatinine, blood flow to the kidney, sodium excretion and urine output. All of these end points have their benefits, but also several shortcomings.

Valproic acid (VPA), an anti-epileptic agent has been shown to have anti-inflammatory and anti-apoptotic properties in ischemic injury [4, 5]. VPA via histone deacetylase inhibition enhances nuclear histone acetylation that increases gene transcription and appears to protect cells from apoptosis through the β catenin survival pathway. The β catenin survival pathway involves serine threonine kinase (Akt), phosphatidylinositide 3-kinase (PI3K) and the interaction of BAD (BCL2-associated death promoter) with BCL2 (B-cell lymphoma 2) proteins. It appears phosphor-Akt phosphorylates BAD, a pro-apoptotic protein. The phosphorylation of BAD enables BCL2 to act as an anti-apoptotic factor and promote cell survival [6]. Thus, it appears VPA has the potential to lead cells towards a survival pathway.

Dexamethasone (Dex) has been shown to ameliorate renal IR injury after 24 hours following a single pre-ischemia dose. The exact mechanism for proximal tubule protection is not known. However, it has been shown to be protective in cardiac and neuronal tissue through a non-genomic pathway, activating endothelial nitric oxide synthase system via the PI3K and Akt pathways [7].

Lipocalin-2 also called neutrophil gelatinase-associated lipocalin (NGAL); osteopontin (bone phosphoprotein); KIM-1 (kidney injury molecule 1) also called TIM-1 (T Cell immunoglobulin and mucin domain containing protein-1) and HAVCR (Hepatitis A Virus Cellular Receptor 1), and albumin are some of the novel potential urine biomarkers of acute kidney injury [8–13]. However, conclusive evidence on their timeline of appearance and disappearance following renal injury is still lacking. DNA microarray analysis for gene expression (mRNA transcripts) is an interesting approach to make an initial assessment of novel markers that appear and /or disappear with renal IR injury and treatment [14].

The need for improved therapeutic measures to mitigate perioperative renal dysfunction and early diagnosis of renal dysfunction encouraged us to perform this study. The objectives of this study were: 1) to evaluate the potential benefits of VPA and Dex in renal protection, and 2) to identify novel early biomarkers and pathway knowledge associated with kidney IR injury and recovery.

Our study, which includes both urine and kidney tissue analyses provides strong support for the use of KIM-1, lipocalin-2, and albumin in the diagnosis of kidney injury at 24 h. Microarray analysis revealed many other candidate biomarkers for early detection of kidney injury in future studies. Furthermore, pathway analysis revealed that the pathological phenotypes in rats treated with VPA was indistinguishable from naïve rats by 120 h, suggesting that VPA is a promising therapy to promote recovery from IR injury in the kidney. Lastly, we identified functionally enriched KEGG (Kyoto Encyclopedia of Genes and Genomes) pathways and transcription factors that provide the basis for more targeted therapies to enhance IR injury recovery in the kidney in future studies.

## Materials and Methods

### Ethics statement

The care and use of the rats were approved by the animal Experiment Ethics Committee of Madigan Army Medical Center. The protocol (No. 212128) was approved by the Madigan Army Medical Center’s Institutional Animal Care and Use Committee (IACUC) on the Ethics of Animal Experiments. All animal experiments were performed according to institutional guidelines with the prior approval by the IACUC. All surgery was performed under anesthesia, and all efforts were made to minimize the animal suffering.

### Animals

Wister rats (200–300 g; male) purchased from Harlan Laboratories were housed in Madigan Army Medical Center animal facility as per the *Guidelines for the Care and Use of Laboratory Animals of the National Institutes of Health*. All animal experiments were performed according to institutional guidelines with the prior approval by the IACUC. Animals were monitored closely soon after surgery and twice daily thereafter for their health and wellbeing. To relieve pain, analgesic buprenorphine (0.03mg/kg) was injected subcutaneously at 12 h intervals for three days post-operatively. We used humane protocol end points in our animal survival study. Animals were sacrificed at pre-determined time points (3, 24 and 120 h) following ischemia-reperfusion procedure. However, if animals showed signs of distress and pain (anorexia, lack of grooming and activity, rapid respiration, a moribund state) not alleviated by analgesics, they were removed from the study and euthanized humanely. The animals were euthanized by injecting Sodium Pentobarbitol (40–80 mg/rat) intraperitoneally.

### Kidney Ischemia-Reperfusion injury model

Rat renal IR injury models have been used to create proximal tubular damage using warm ischemia [15]. Previously published rat kidney IR injury model [16] was used, but >80% of the rats died within three days post-IR (n = 9). The procedure was slightly modified by decreasing ischemia time and performing right nephrectomy following reperfusion of the ischemic left kidney allowing rat survival ≥5 days. The model was designed with the objective of creating underlying decreased nephron mass and rat survival of at least five days to assess the perioperative renal dysfunction, as well as recovery. Rats were premedicated with VPA or Dex, prior to ischemia induction in an attempt to protect the renal parenchyma.

Briefly, rats were anesthetized by isoflurane inhalation induction followed by ketamine (~70 mg/kg) and xylazine (~6 mg/kg) administration intraperitoneally. Rats were premedicated with Valproic acid [17] at 150 mg/kg, Dex at 3 mg/kg [7] or Vehicle (saline control) intraperitoneally thirty minutes prior to surgery. A sterile surgical site was prepared, and a midline incision was performed to access the abdominal cavity. The left kidney was located, and its renal artery was clamped using an atraumatic microaneurysm clamp. Renal ischemia was confirmed by the kidney color which was pale. The abdomen was approximated to prevent dehydration and maintain body temperature. Animal was kept on heat controlled thermal pads to maintain body temperature throughout the procedure. Following 45 min (minutes) of warm ischemia the abdomen was reopened and the renal clamp was removed. Change in the kidney color (pale to bright red) confirmed proper reperfusion. Reperfusion was quickly followed by a right nephrectomy. Finally, the abdomen was closed by suturing muscles and /or fascia with 6-O prolene, and the skin was closed with 4-O nylon and clips.

### Experimental design

**Fig 1:**
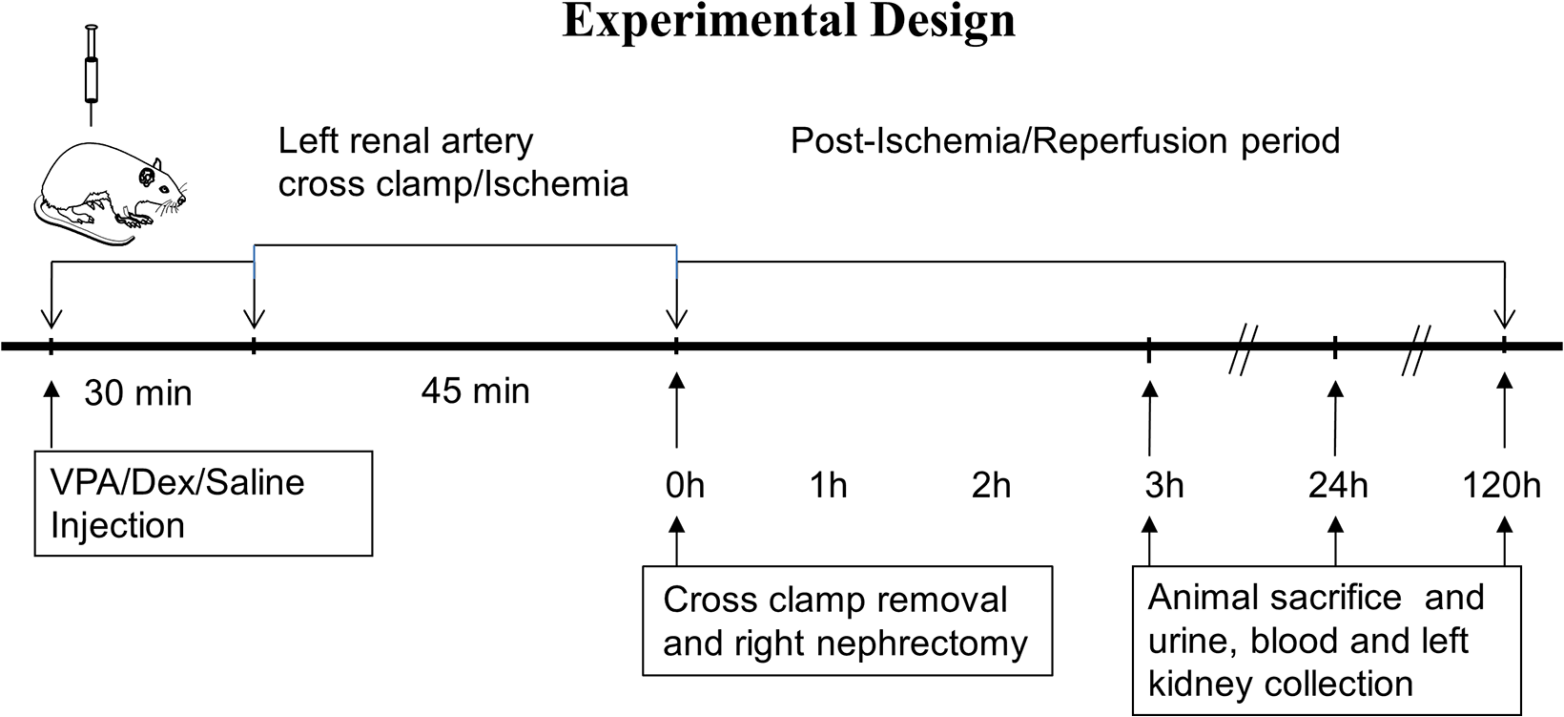
Experimental design. Lewis rats were pre-medicated with Valproic acid (VPA; 150 mg/kg body weight) or Dexamethasone (Dex; 3 mg/kg/body weight) or saline (Vehicle control) intraperitoneally 30 minutes (min) prior to cross clamping left renal artery and inducing left renal ischemia. The cross clamp was removed after 45 min allowing kidney reperfusion, and at the same time a right nephrectomy was performed. Animals were sacrificed at 3, 24 and 120 hours post ischemia-reperfusion. Left kidney, blood and urine were collected for cellular and molecular analyses.

The study included three experimental groups. Group A, VPA treatment; Group B, Dex treatment; and Group C, No treatment (Vehicle i.e., saline control). Treatments were administered before inducing ischemia. Rats underwent left renal ischemia for 45 min, followed by the removal of renal artery clamp (to allow reperfusion) and right nephrectomy. Following reperfusion, animals were sacrificed at 3, 24 or 120 h (h = hour/s; n = 8/group). In the 3 h group, rats were maintained under anesthesia after surgery until sacrifice. In the 24 and 120 h groups, the rats were recovered and returned to the cages for normal housing. Analgesic buprenorphine (0.03 mg/kg) was injected subcutaneously at 12 h intervals for three days post-operatively. After animal sacrifice, urine, blood, and kidney were collected for kidney functional biomarker assays, histology and/or molecular analyses. Kidneys and urine samples collected from normal (naïve) uninjured/untreated animals (n = 5) immediately following general anesthesia and sacrifice were used for baseline measurements (Fig 1).

### Specimen collection and storage

After animal sacrifice, the left kidney was excised and bisected longitudinally. One half was stored in 10% phosphate buffered formalin fixation solution for histopathology, and the other half (cut into 2–4 mm pieces) was snap frozen in liquid nitrogen and stored at -80°C for molecular analyses. Cystocentesis was performed to obtain urine samples from each rat at the time of sacrifice. The urine samples were snap-frozen and stored at -80°C for the analysis of kidney injury biomarkers at a later time. Blood samples were collected from left renal vein and analyzed immediately for kidney functional biomarkers (blood urea nitrogen [BUN] and creatinine).

### Blood and urine biomarkers of kidney injury/function

Blood biomarkers for kidney injury/function were measured using HESKA rat kidney test panels, as per manufacturer’s instructions, using a Dri-chem analyzer (HESKA Corporation, Loveland, Colorado). Kidney panels included BUN and creatinine measurements. Briefly, the kidney panel slides (stored at 4°C) were warmed to room temperature for five min prior to use. Blood plasma samples were loaded in 0.5 mL tubes in the Dri-chem analyzer. Predefined protocol/assay was selected to run the sample and collect data.

The urine bio-markers albumin, KIM-1, lipocalin-2 and osteopontin concentrations were analyzed using the Multiplex Immunoassay System kit (Rat Kidney Injury Panel-1; Meso Scale Discovery, Gaithersburg, MD) as per manufacturer’s instructions. Briefly, 150 μL/well of blocker ‘A’ solution was added and incubated at room temperature for 1 h with vigorous shaking (~600 rpm). The wells were washed with PBS-T three times. Fifty microliters of the sample (controls, unknowns or standards) premixed with 1x albumin tracer was added to each well and incubated at room temperature for 2 h with vigorous shaking. After washing plates as before, 1x detection antibody solution (25 μL/well) was added and the plates were incubated at room temperature for 2 h with vigorous shaking. Again plates were washed as before, and 150 μL of Read Buffer-T was added. The plates were analyzed on the Sector Imager that provided quantitative measurement of analytes in the sample based on the intensity of light emitted.

### Histopathology

Histopathologic examination of formalin fixed paraffin embedded, 4–5 μm thick longitudinal cross-sections of the left kidney stained with Gill’s modified Hematoxylin and Eosin was performed. Specimens were examined in a blinded manner by two pathologists independently under light microscopy as described previously [18–20] with slight modifications. Briefly, three high power fields (at 400x magnification, representing approximately 50 tubules) from the cortex and outer medulla of each kidney were examined and graded for predominant injury patterns including ischemic changes (injury), tubular necrosis, and regenerative changes. Ischemic changes were defined as nuclear condensation, cytoplasmic eosinophilia, individual cell necrosis, and tubular dilation. Tubular necrosis was characterized by confluent cell necrosis or sloughing of the tubular epithelium. Regenerative changes included tubular dilation, cytoplasmic basophilia and contraction of the cytoplasm, as well as vesicular chromatin with nucleoli. Collectively, kidney injury and regeneration were graded (0 to 4) based on the mean percentage of affected tubules: 0, none; 1, <25%; 2, ≥25% but <50%, 3, ≥50% but <75%; and 4, ≥75%-100% [19].

### Total RNA isolation

Kidney tissues snap frozen and stored at -80°C were used for RNA isolation. Tissue piece (100 mg) was transferred to a DNA/RNA-free round bottom tube containing CHAPS buffer (Cat. No. 90436, Millipore Co., CA) and gently homogenized (TEKMAR Tissuemizer Co., Cincinnati, OH) for 10–20 s (seconds). The homogenized tissue was kept in ice for 20 min followed by centrifugation (12,000 x g for 20 min). The supernatant (200 μL) was mixed with 400 μL of RNA lysis buffer (High Pure RNA Isolation kit, Cat. No.11 828 665001, Roche Diagnostics, Mannheim, Germany) and the procedure was continued as per manufacturer’s instructions. The isolated RNA was assessed for purity and quantity by spectrophotometry (Nanodrop ND-100, Nanodrop technologies, Inc., Wilmington, DE). A ratio >2 OD @ 260 nm/280 nm was considered high quality RNA for further studies.

### Real-time quantitative polymerase chain reaction

Gene expression (mRNA transcript level) of pro-inflammatory cytokines (IL1β and IL6) and anti-apoptotic molecule (BCL2) was measured in the total RNA by reverse transcription using relative quantitative PCR (Rel*q*PCR). The PCR was performed in 96-well LightCycler480 platform using the one-shot method (Roche Applied Sciences, Mannheim, Germany) and Hydrolysis Kit (Cat. No. 04991885001, Roche Applied Sciences), as per the manufacturer’s recommendation. Briefly, primers for all selected genes were constructed, and specific probes were chosen using Roche’s Universal Probe Library Assay Design Center software (https://www.roche-applied-science.com). The primers and probes used were: 1) IL1β, Forward 5’-GCTGACAGACCCCAAAAGAT-3’, Reverse 5’-AGCTGGATGCTCTCATCTGG-3’, probe 117, amplicon size 72 bp; 2) IL6, Forward 5’-GCCCTTCAGGAACAGCTATG-3’, Reverse 5’-GCAGTGGCTGTCAACAACA-3’, probe 127, amplicon size 86 bp; 3) BCL2, Forward 5’-GTACCTGAACCGGCATCTG-3’, Reverse 5’-GGGGCCATATAGTTCCACAA-3’, probe 75; amplicon size 76 bp; and 4) rat β-Actin, commercial primers and probe (Cat No. 05046203001, Roche Applied Sciences, US).

The PCR was performed using total RNA (100 ng) template, 0.5 μM of primers, and 0.25 μM of hydrolysis probe in triplicates. Thermocycling conditions included a 30 min reverse transcription at 63°C, and initial denaturation for 30 s at 95°C; this was followed by 45 cycles of denaturation (95°C for 10 s), annealing (60°C for 30 s) and extension (72°C for 1 s), and a final cooling step at 4°C. Each sample was run in triplicates using specific primers for the target gene and for β-Actin (internal control), a housekeeping gene. Gene transcripts were normalized to β-Actin (Target gene: β-Actin). The gene expressions are presented as Target gene: β-Actin ratio and /or fold increase or decrease in VPA and Dex treated groups compared to Vehicle control group. If the ratio of gene expression (i.e., ratio of VPA or Dex treated to vehicle control group) is >1.1 or <0.9, then it is designated as “upregulation” or “downregulation”, respectively; ratio between 0.9 and 1.1 is designated as “unchanged.” The mean values of gene expression between control and VPA or Dex groups (n≥4 animals) were compared using student ‘t’ test for statistical significance (P<0.05).

### Microarray assay and data analysis

The kidney tissues snap-frozen and stored at -80°C were used for mRNA microarray analyses as previously described [21]. Total RNA was isolated using the miRNeasy 96 kit (Qiagen, Cat No 217061, Valencia, CA), according to the manufacturer’s instructions. Quality and quantity of RNA were evaluated with a 2100 Bioanalyzer (Cat. No. 62947, Agilent Technologies, Santa Clara, CA), using the Agilent RNA 6000 Nano Reagents (Cat. No. 5067–1511, Agilent Technologies,), and a multi-well Nanodrop 8000 spectrophotometer (Cat. No. 1464, Scientific, Waltham, MA).

#### Affymetrix gene array

RNA was diluted to 50 ng/μL, and synthesized to cRNA using the Ambion Whole Transcript (WT) Expression Kit (Ambion, Cat. No. 4411974, Austin, TX) according to the manufacturer’s instructions. The cRNA was quantified by NanoDrop and 455 ng/μL was used as a template to synthesize cDNA. The samples were then fragmented using the GeneChip WT Terminal Labeling Kit (Cat. No. 901524, Affymetrix, Santa Clara, CA), labeled, and prepared with the GeneTitan Hybridization, Wash and Stain Kit for whole-transcript array plates (Affymetrix, Cat. No. 901622), according to the manufacturer’s instructions. Samples were then applied to the Rat Gene 1.1 ST 16 Array Plate or 24 Array Plate (Affyemetrix, Cat. No. 901432, 901431, respectively) and placed in the GeneTitan System (Affymetrix, Cat. No. D0101330), according to the manufacturer’s recommendations. Of the 44 arrays, two did not hybridize appropriately and did not pass the GeneTitan scanning QC, and were excluded from further analysis.

#### Gene array data analysis

Kidney DNA microarray data were imported into Partek, and Two-Way ANOVA with contrasts at 3, 24 and 120 h was conducted as described previously [22]. Raw CEL files were normalized using iterPLIER in Expression Console, which discarded feature sets that performed poorly, similar to the previous report [23]. The resulting CHP files were imported into Partek Discovery Suite (Partek, Inc., St. Louis, MO). Affymetrix library files included all available reference files related to RaGene-1_1-st-v1.na33.2.rn4.probeset.csv. A variance-stabilizing transformation (PLIER+16) was applied to the raw intensity (Guide to Probe Logarithmic Intensity Error [PLIER] Estimation) to resolve signal variance following the PLIER analysis. The standard deviation of each transcript ID was determined, the lower 50% were removed, and the remaining 50% of transcript IDs were used as background for pathway analysis. The low variance criteria from Bourgon et al. [24] was implemented by computing and sorting the expression variance of each gene over the complete condition set, then removing the low-variance genes (lower half). To determine differentially expressed genes (DEGs), a Three-Way ANOVA was conducted and gene lists were generated by applying a two-fold change cut off and *P*-value with Benjamini-Hochberg False Discovery Rate (FDR) multiple testings (<0.05) correction [25, 26]. When few DEGs were identified, DEGs with uncorrected *P*-values were determined for the purpose of pathway analysis.

#### Gene enrichment analysis

Gene lists were examined with the Database of Annotation, Visualization and Integrated Discovery (DAVID) [27]. Enriched KEGG (Kyoto Encyclopedia of Genes and Genomes) pathways and functional annotation clusters (FACs) were determined using default settings. The most significant biological processes, molecular functions and cellular components from functionally associated clusters with an enrichment score >2.0 were represented and examined. Gene lists were consolidated and sub-categorized to account for redundant annotation terms comprising FACs. For representation purposes, unique transcripts were consolidated from similar FACs.

The DNA microarray data have been deposited in NCBI’s Gene Expression Omnibus (GEO) repository and are accessible through Accession No. GSE58438 (http://www.ncbi.nlm.nih.gov/geo/). Activation of transcription factors were determined using www.TFactS.org. as described previously by Essaghir and Co-Workers[28]. Predicted transcription factors (TFs) were considered for all treatment conditions if the random control (RC %) was less than 2%, the *P*-value was less than 0.01, and FDR was less than 0.01, with at least 3 genes identified. For the TfactS analysis, the *P*-value was computed using the Fisher’s test with the Hypergeometric formula, the E-value is the number of tests performed times the *P*-value, the Q-value is the minimum FDR that can be obtained when calling a TG (Target Gene) significant, and the RC is the percentage of which a TF is called significant under a certain E-value threshold after a random simulation of the gene lists.

### Statistical analysis

The data were analyzed for statistical significance and descriptive statistics using *PASW Statistics version 18* software program, Partek Discovery Suite or www.TFactS.org where appropriate. For biochemical analyses, the mean values between two groups were compared by *t* test and among ≥ three groups by One-Way ANOVA with post-hoc (Bonferroni or LSD [Fisher’s Least Square Difference]) correction. All *P*-values presented <0.05 are considered statistically significant, and are two-tailed values.

## Results

### Kidney Ischemia-Reperfusion injury model

In this study, with slight procedural modifications, the rat survival was ≥5 days in 85% of the animals studied. The modifications included reducing left renal ischemia time to 45 minutes, isolated left renal artery clamping, and right nephrectomy following reperfusion.

### Blood biomarkers of kidney function

**Fig 2:**
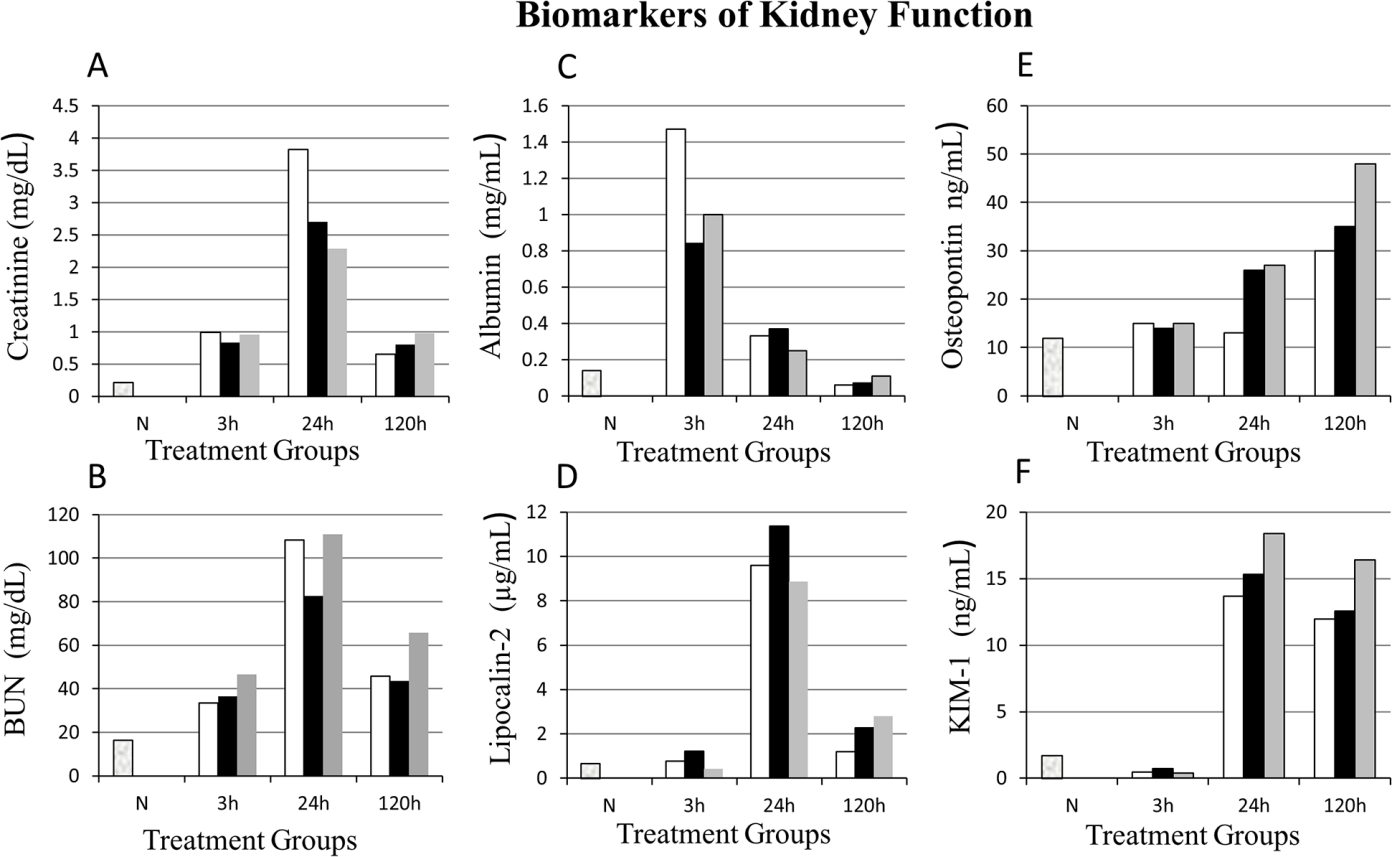
Kidney functional biomarker analysis. X-axis represents treatment groups (n = 8/group): Vehicle control (white bars □), Valproic Acid (VPA; black bars ■), and Dexamethasone (grey bars ■). N, Normal (naïve) uninjured and untreated rats (n = 5). Y-axis represents concentration of each biomarker in the blood (creatinine, blood urea nitrogen [BUN]) and urine (albumin, lipocalin-2, osteopontin, kidney injury molecule-1[KIM-1]). In VPA treated animals albumin level were markedly lower at three hours (h) while the creatinine and BUN values were lower at 24 h post ischemia-reperfusion (IR) compared to vehicle controls. However, lipocalin-2 and KIM-1 were higher at 24 h in all the groups (VPA, Dex, Vehicle) studied. See Tables 1 & 2 for statistical significance between treatment groups and with time post ischemia-reperfusion.

Blood plasma creatinine (mg/dL), a commonly used marker to evaluate kidney function clinically was lower in the VPA (2.7±1.8) and Dex (2.3±1.2) treatment groups compared to Vehicle control (no treatment) group (3.8±0.5) at 24 h reperfusion (Tables 1 & 2; Fig 2). By five days post-IR, plasma creatinine levels came back to nearly normal. BUN (mg/dL) was elevated in all IR groups (≥ 33) compared to naive animals (16.5±2.4). However, there was no difference in BUN among IR groups with the exception of Dex at 3 h, which was higher (P<0.05) than the Vehicle control (Table 2; Fig 2).

**Table 1:** Comparisons of blood plasma and urine biomarkers (mean ± standard deviation) between time points (post kidney ischemia-reperfusion) in different treatment groups.*

	Nor-mal/ Naive	Vehicle (Control)			VPA			Dex		
**Marker**		3h	24h	120h	3h	24h	120	3h	24h	120h
**Creatinine**	0.22±	0.99±	3.82±	0.65±	0.83±	2.7±	0.8±	0.96±	2.29±	0.97±
**(mg/dL)**	0.04	0.13^a^	0.46^b^	0.32^a^	0.24^a^	1.78^b^	0.83^a^	0.15^a^	1.22^b^	0.48^a^
**BUN**	16.50±	33.56±	108.36±	45.71±	36.46±	82.69±	43.57±	46.63±	110.87±	65.62±
**(mg/dL)**	2.43	3.35^a^	8.92^b^	19.10^a^	5.25^a^	47.64^b^	31.80^a,b^	8.48^a^	44.75^b^	33.70^a,b^
**Albumin**	0.14±	1.47±	0.33±	0.06±	0.84±	0.37±	0.07±	1.04±	0.25±	±0.11
**(mg/mL)**	0.26	0.10^a^	0.19^b^	0.06^b^	0.62^a^	0.31^a,b^	0.03^b^	0.73^a^	0.10^b^	0.07^b^
**Lipocalin-2**	0.67±	0.77±	9.61±	1.19±	1.21±	11.36±	2.27±	0.42±	8.86±	2.79±
**(μg/mL)**	0.29	0.31^a^	4.39^b^	0.61^a^	0.32^a^	3.83^b^	.86^a^	0.15^a^	1.35^b^	1.69^c^
**Osteopontin**	12±	15±	13±	30±	14±	26±	35±	15±	27±	48±
**(ng/mL)**	12	10^a^	9^a^	29^a^	7^a^	19^a,b^	11^b^	14^a^	20^a^	31^b^
**KIM-1**	1.7±	0.5±	13.7±	11.9±	0.7±	15.3±	12.6±	0.4±	18.7±	16.4±
**(ng/mL)**	0.9	0.2^a^	9.9^b^	11.5^b^	0.4^a^	11.4^b^	11.1^b^	0.4^a^	13.0^a^	6.2^a^

*Creatinine and BUN (Blood Urea Nitrogen) were measured in blood plasma

Albumin, Lipocalin-2, Osteopontin, and KIM-1 (Kidney Injury Molecule-1) were measured in urine. **Normal/Naive,** plasma or urine drawn from naïve uninjured/untreated Wister rats served as base line. **VPA** (Valproic Acid), **Dex** (Dexamethasone) and **Vehicle** (saline control) treated groups at 3, 24 and 120 hour (h) reperfusion. Means with at least one common superscript (a, b or c) between 3, 24 or 120 h within each group (Vehicle, VPA or Dex) did not vary significantly (P>0.05).

**Table 2:** Comparisons of blood plasma and urine biomarkers (mean ± standard deviation) between treatment groups at 3, 24, and 120 hours post kidney ischemia-reperfusion (IR).*

	Nor-mal/ Naive	3h post-IR			24h post-IR			120h post-IR		
Marker		Vehicle	VPA	Dex	Vehicle	VPA	Dex	Vehicle	VPA	Dex
**Creatinine**	0.22±	0.99±	0.83±	0.96±	3.82±	2.7±	2.29±	0.65±	0.8±	0.97±
**(mg/dL)**	0.04	0.13^a^	0.24^a^	0.15^a^	0.46^a^	1.78^a,b^	1.22^b^	0.32^a^	0.83^a^	0.48^a^
**BUN**	16.50±	33.56±	36.46±	46.63±	108.36±	82.69±	110.87±	45.71±	43.57±	65.62±
**(mg/dL)**	2.43	3.35^a^	5.25^a^	8.48^b^	8.92^a^	47.64^a^	44.75^a^	19.10^a^	31.80^a^	33.70^a^
**Albumin**	0.14±	1.47±	0.84±	1.04±	0.33±	0.37±	0.25±	0.06±	0.07±	0.11±
**(mg/mL)**	0.26	0.10^a^	0.62^a^	0.73^a^	0.19^a^	0.31^a^	0.10^a^	0.06^a^	0.03^a^	0.07^a^
**Lipocalin-2**	0.67±	0.77±	1.21±	0.42±	9.61±	11.36±	8.86±	1.19±	2.27±	2.79±
**(μg/mL)**	0.29	0.31^a^	0.32^b^	0.15^c^	4.39^a^	3.83^a^	1.35^a^	0.61^a^	0.86^a,b^	1.69^b^
**Osteopontin**	12±	15±	14±	15±	13±	26±	27±	30±	35±	48±
**(ng/mL)**	12	10^a^	7^a^	14^a^	9^a^	19^a^	20^a^	29^a^	11^a^	31^a^
**KIM-1**	1.7±	0.5±	0.7±	0.4±	13.7±	15.3±	18.7±	11.9±	12.6±	16.4±
**(ng/mL)**	0.9	0.2^a^	0.4^a^	0.4^a^	9.9^a^	11.4^a^	13.0^a^	11.5^a^	11.1^a^	6.2^a^

*Creatinine and BUN (Blood Urea Nitrogen) were measured in blood plasma

Albumin, Lipocalin-2, Osteopontin, and KIM-1 (Kidney Injury Molecule-1) were measured in urine. **Normal/Naive**, blood plasma or urine drawn from naïve uninjured/untreated Wister rats served as base line. **VPA** (Valproic Acid), **Dex** (Dexamethasone) and **Vehicle** (saline control) treated groups at 3, 24 and 120 hour (h) reperfusion. Means with at least one common superscript (a, b or c) between Vehicle (control), VPA or Dex within each group (3, 24 or 120 h) did not vary significantly (P>0.05).

### Urine biomarkers of kidney function

At 3 h post-IR, the urine albumin (mg/mL) was lower but not significant (P>0.05) in VPA (0.84±0.62) and Dex (1.04±0.73) compared to Vehicle control (1.47±1.02) group. The albumin levels further decreased in all groups steadily at 24 and 120 h (Table 2, Fig 2). Lipocalin-2 levels were significantly (P<0.05) higher in VPA compared to Vehicle control at 3 h (Table 2). At 24 h, osteopontin was higher in VPA and Dex compared to Vehicle but not statistically significant (P>0.05). At 120 h after IR injury, lipocalin-2 was higher in VPA and Dex compared to Vehicle control group (Table 2). Interestingly, KIM-1 was reduced at 3 h post-IR in all groups compared to naïve rats, but significantly (P<0.05) increased at 24 and 120 h (Table 2). In summary, compared to normal levels in naive rats, at 3 h post-IR, albumin was a good marker of injury; while at 24 h post-IR lipocalin-2 and KIM-1 appear to be better markers of injury. At 120 h post-IR, KIM-1 and osteopontin continued to be significantly (P<0.05) higher than in naïve rats (Tables 1 & 2; Fig 2).

### Histopathology

**Fig 3:**
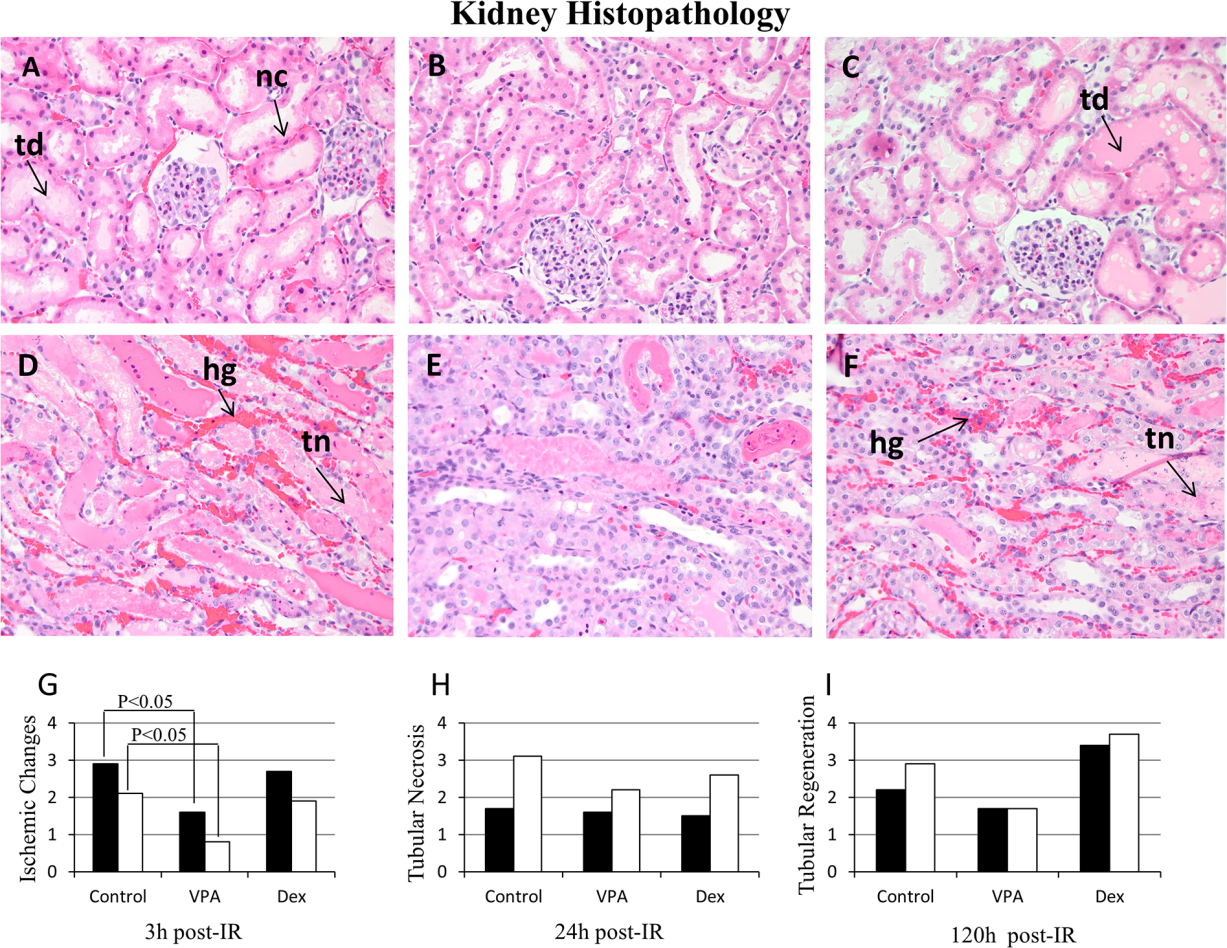
Histopathology of hematoxylin and eosin stained kidney sections. A, B, C = Renal cortex at 3 hours (h) post ischemia-reperfusion (IR); D, E, F = Renal outer medulla at 24 h post-IR; A, D = Vehicle (saline control); B, E = Valproic acid (VPA); C, F = Dexamethasone (Dex) treated animals. Three high power fields (400x) representing approximately 50 tubules from cortex and outer medulla of each kidney were evaluated for ischemic changes (injury), tubular necrosis and regenerative changes. Collectively kidney injury and regeneration were graded (0–4) based on the mean percentage of tubules affected: 0, None; 1, <25%; 2, ≥25 but <50%; 3, ≥50 but <75%; 4, >75–100%. Ischemic changes included nuclear condensation **(nc)**, cytoplasmic eosinophilia, individual cell necrosis and tubular dilation **(td)**; tubular necrosis **(tn)** included confluent cell necrosis or sloughing of the tubular epithelium; and regenerative changes included tubular dilation, cytoplasmic basophilia and contraction of the cytoplasm, as well as vesicular chromatin with nucleoli. Hemorrhage **(hg)** was predominant in the vehicle control group. G, H, I = represent Histopathology quantification: renal cortex (black bars ■) and renal outer medulla (white bars □). The histologic injury score was significantly (P<0.05) lower in the VPA treated group compared to the Vehicle control at 3 h post-IR (see Table 3).

Ischemic changes, tubular necrosis and tubular regeneration were predominant at 3, 24 and 120 h post-IR, respectively. The histopathologic score for ischemic changes at 3 h post-IR in the renal cortex was significantly (P<0.05) lower (1.57±1.51) in VPA compared to Vehicle control (2.87±1.12) group; also, it was significantly lower in the medulla (0.86±0.9) compared to Vehicle control group (2.12±0.83) (Fig 3G; Table 3). The ischemic changes that included nuclear condensation, cytoplasmic eosinophilia, individual cell necrosis, and tubular dilation were less severe in both renal cortex and medulla of VPA treated animals. At 24 h post-IR, tubular necrosis in the medulla was markedly reduced in VPA (2.25±1.28) compared to Vehicle control group (3.12 ±0.64) (Fig 3H; Table 3). The tubular necrosis that included confluent cell necrosis or sloughing of the tubular epithelium was less severe with VPA therapy. In Dex treated animals, ischemic changes at 3 h post-IR and tubular necrosis at 24 h post-IR did not differ significantly (P>0.05) from the Vehicle control group; however, tubular regeneration was markedly higher in Dex treated group at 120 h post-IR compared to Vehicle control and VPA groups (Fig 3I; Table 3). The regenerative changes that included tubular dilation, cytoplasmic basophilia, and contraction of the cytoplasm, as well as vesicular chromatin with nucleoli were improved at 120 h in Dex treated animals compared to Vehicle or VPA groups. Hemorrhage was predominant in the Vehicle treated group compared to VPA or Dex group. Histopathology (H&E stain) microphotographs and data at 3 h and 24 h post-IR in VPA, Dex, and Vehicle groups are presented in Fig 3A–3F and Table 3.

**Table 3:** Histopathologic scores (Mean± SD) of renal cortex and outer medulla at 3, 24 and 120 hours (h) post ischemia-reperfusion (IR) in Valproic Acid (VPA) and Dexamethasone (Dex) treated animals.

Group	Ischemic Changes at 3h post-IR		Tubular Necrosis at 24h post-IR		Tubular Regeneration at 120h post-IR	
	Cortex	Medulla	Cortex	Medulla	Cortex	Medulla
**Vehicle (Control)**	2.87±	2.12±	1.75±	3.12±	2.25±	2.87±
	1.12^a,1^	0.83^a,2^	1.03^a,1^	0.64^a,2^	1.39^a,1^	1.46^a,2^
**VPA**	1.57±	0.86±	1.62±	2.25±	1.75±	1.75±
	1.51^b,1^	0.9^b,1^	0.74^a,1^	1.28^a,1^	2.06^a,1^	2.06^a,1^
**Dex**	2.75±	1.87±	1.5±	2.62±	3.43±	3.71±
	1.03^a,b, 1^	0.83^a,2^	0.92^a,1^	0.92^a,2^	0.53^a,1^	0.49^b,1^

Mean values within columns among treatment groups with at least one common ***superscript letter (a or b)*** did not vary significantly (P>0.05). Also, mean values in rows between cortex and medulla (within 3h, 24h or 120h post-IR) groups with a common ***superscript number*** did not vary significantly (P>0.05). Vehicle (saline) served as a control. N = 8/sub-group (3, 24 or 120 h).

### Inflammatory cytokine and anti-apoptotic molecule BCL2 expression

**Fig 4:**
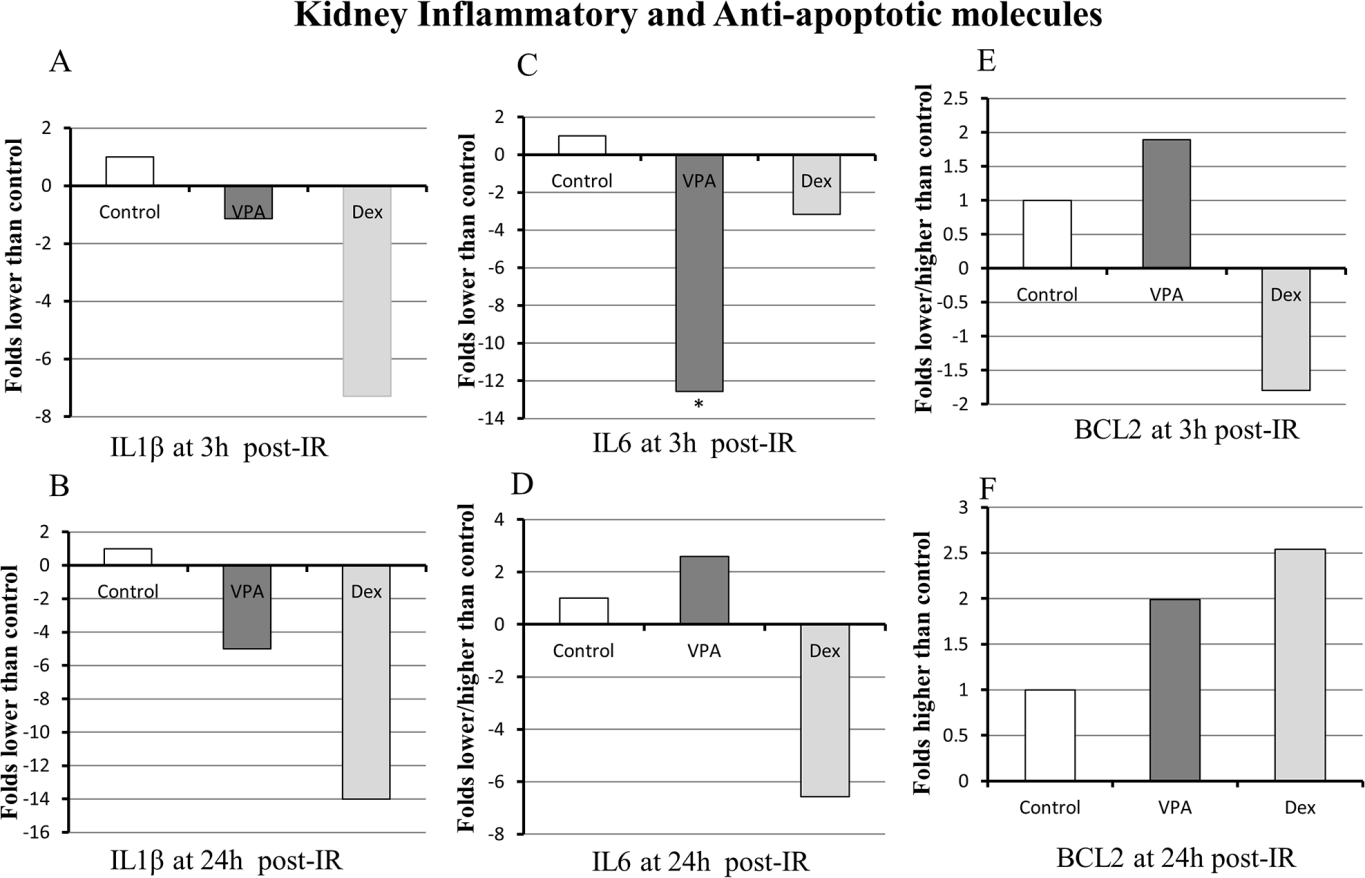
Inflammatory and anti-apoptotic molecule expression as determined by the reverse transcription-polymerase chain reaction (RT-PCR). The inflammatory cytokine IL1β and/or IL6 were markedly downregulated in Valproic acid (VPA) treated animals compared to Vehicle treated controls at 3 hours (h) and 24 h post ischemia-reperfusion (IR). The anti-apoptotic molecule BCL2 was upregulated at 3 and 24 h post-IR in VPA treated group. The RT-PCR was performed in triplicates using RNA derived from kidney samples (n≥ 4 animals from each group). Gene transcripts were normalized to β-Actin (Target gene: β-Actin). The gene expressions are presented as fold increase or decrease in VPA and Dex treated groups compared to Vehicle control group. If the ratio of gene expression (i.e., ratio of VPA or Dex treated to vehicle control group) is >1.1 or <0.9, then it is designated as “upregulation” or “downregulation”, respectively. The mean values of gene expression between control and VPA or Dex groups were compared using student ‘t’ test and ‘*’ notation over or under the bar represents statistical significance (P<0.05).

IL1β expression in the kidney was 1-fold and 5-fold lower in VPA treated group compared to Vehicle control at 3 h and 24 h post-IR, respectively; in Dex treated group it was 7-fold and 14-fold lower compared to Vehicle control (Fig 4A and 4B). IL6 expression in the kidney was 12-fold lower (P<0.05) in VPA compared to Vehicle control at 3 h post-IR; in Dex treated group it was 3-fold and 7-fold lower at 3 h and 24 h post-IR, respectively, compared to Vehicle control (Fig 4C and 4D). The anti-apoptotic molecule BCL2 expression in the VPA treated kidney was about 2-fold higher than Vehicle control at 3 h and 24 h post-IR; in Dex treated group BCL2 was increased (2.5-fold) at 24 h post-IR compared to Vehicle control (Fig 4E and 4F). Overall, IL1β expression was lower in VPA and Dex treated animals compared to untreated controls at 3 and 24 h post-IR; IL6 expression was lower in VPA and Dex at 3 h post-IR; and the BCL2 expression was higher in VPA and Dex at 24 h post-IR.

### DNA microarray analysis of gene expression

**Fig 5:**
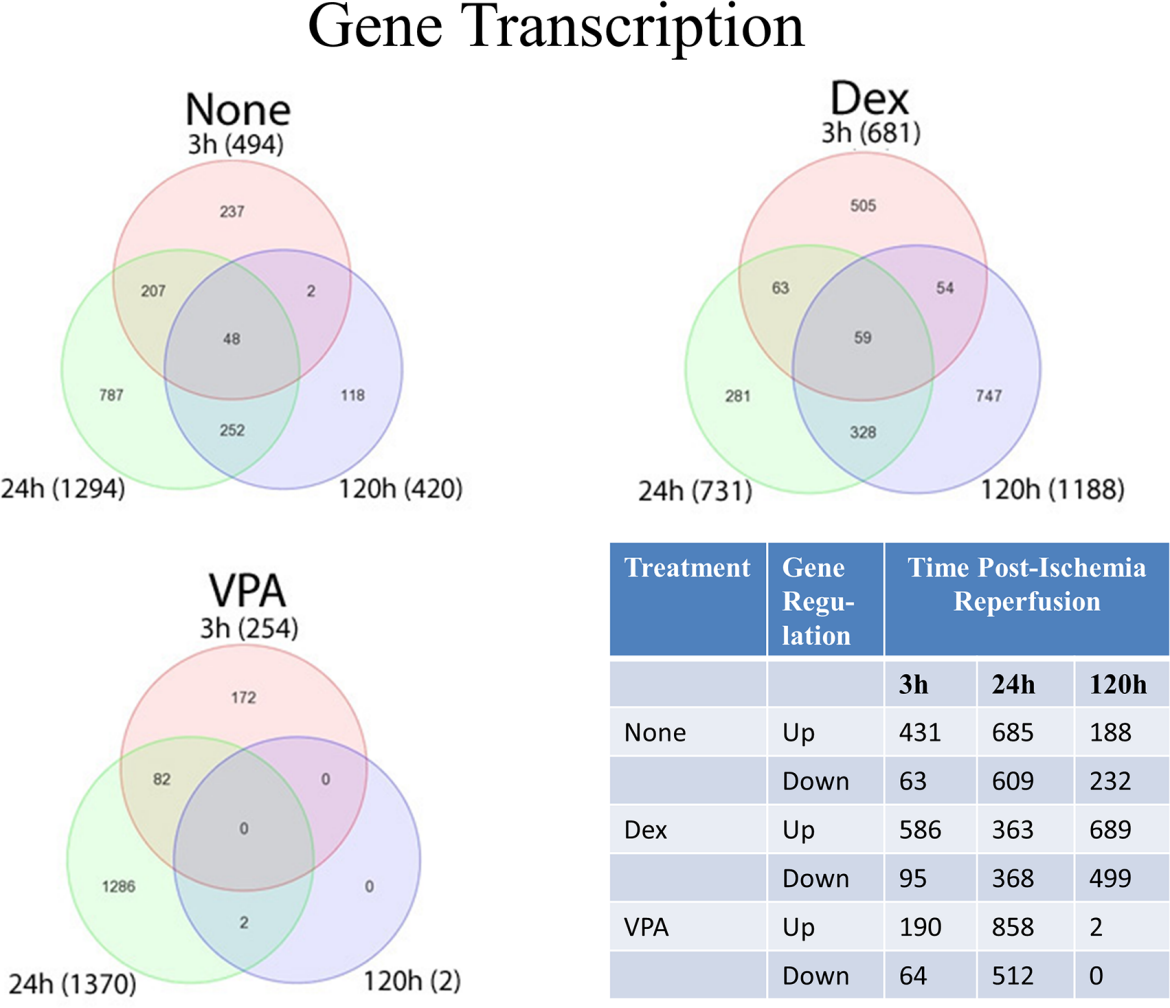
Gene transcription analysis as determined by Affymetrix Gene Array Technology. The Venn diagrams presented show the number of transcriptomes expressed and overlaps in the kidneys of animals treated with valproic acid (VPA), dexamethasone (Dex) or untreated (None; Vehicle control) at 3, 24 and 120 hours (h) post ischemia-reperfusion (IR). All relative changes in gene expression are derived from comparison with naïve rats. Fewer genes were up-regulated in the VPA group compared to untreated Vehicle control group at 3 h post-IR. At 120 h post-IR only two genes were upregulated and none was downregulated in VPA treated animals. Specific gene expression information is presented in **S1–S6 Tables**. The DNA microarray data have been deposited in NCBI’s Gene Expression Omnibus (GEO) repository and are accessible through Accession No. GSE58438 (http://www.ncbi.nlm.nih.gov/geo/).

While histological evidence of injury was no longer evident at 120 h in animals treated with VPA, urinary levels of kidney injury markers remained elevated (i.e. Osteopontin and KIM-1). Thus, we decided to evaluate these animals at the transcript level to determine what other markers of injury may be elevated and what, if any, pathways were enriched among the experimental groups to help guide future studies. Differential gene expression in the kidney was determined by Three-Way ANOVA with contrasts. All groups (Vehicle, Dex, or VPA) were compared to naïve (uninjured and untreated) animals at 3, 24, and 120 h. The totals of unique genes found to be upregulated (increased) or downregulated (decreased) compared to levels in normal (naïve) animals by at least two-fold and less than 0.05 false discovery rate (FDR) is summarized in Fig 5. While all groups showed hundreds of changes in gene expression at 3 h and 24 h, only two genes were upregulated (*Clec4a2 and Lox*) at 120 h in animals that were treated with VPA, indicating that VPA animals were virtually indistinguishable from naïve rats. All relative changes in gene expression were derived from comparison with naïve rats.

Since we are interested in identifying novel early biomarkers of kidney injury, expression analysis of the top twenty upregulated and downregulated genes are presented in S1 and S2 Tables, and the full Differentially Expressed Gene (DEG) lists are in S3 Table. Stress induced genes, such as *Hspa1b*, *Hspb1* were upregulated as early as 3 h post-IR in kidneys of all treatment groups. Major regulators of apoptosis were also increased, such as *Atf3*, *Hmox1*, and *Zfand2a*. In agreement with the urinary markers of kidney injury, *Havrc1* (KIM-1) was moderately increased in Vehicle and Dex treated animals, and even lower in VPA treated animals. By 24 h, *Havrc1* and *Lcn2* (Lipocalin-2) were the highest upregulated genes in Vehicle, Dex, and VPA, by +47.1, +56.2, +87.4 fold and +14.7, +16.2, +16.4 fold, respectively. Further, *Timp1* (tissue inhibitor of metalloproteinases, a well-known biomarker of organ injury) was upregulated in all groups at 24 h, which together with *Havcr1* persisted until 120 h, except in VPA treated animals. On the other hand, *A2m*, which is known to increase in nephrotic syndrome and leakage of tubules, was only upregulated in the Vehicle and Dex groups at 24 and 120 h, but not VPA at any time point. Although urinary markers demonstrated a decrease in IL1β and IL6, inflammatory mediators such as *IL19* and *IL24* expressions were both upregulated at 120 h in Vehicle and Dex groups. In VPA, however, both of these cytokines were significantly upregulated as early as 24 h. At 120 h post-IR, gene microarray data demonstrated the upregulation of only two genes *Lox* (Lysyl oxidase) and *CLec4a2* (C-type Lectin domain 4 family 4 member a2; Dendritic cell immunoreceptor).

Gene transcription analysis presented in the Venn diagram (Fig 5) shows the number of transcriptomes expressed and overlaps in the kidneys of animals treated with VPA, Dex and Vehicle groups at 3, 24 and 120 h post-ischemia reperfusion. All relative changes in gene expression were derived from comparison with naïve rats. In the vehicle treated group, at 3 h, only 63 genes were downregulated, whereas, at 24 and 120 h, 609 and 232 genes were downregulated, respectively (Fig 5). A large number of transporters (solute carrier organic anion and cation) and voltage-dependent calcium channel associated gene expressions were downregulated at 24 h and 120 h. Interestingly, at 3 h *cd180* (a negative regulator of toll-like receptor 4 [TLR4]-mediated inflammation) and *Tril* (TRL4 interactor with leucine-rich repeats) were downregulated (S2 Table). At 120 h post-IR only two genes were upregulated and none was downregulated in VPA treated animals. However, several genes were upregulated and downregulated at 120 h post-IR in both Vehicle (no treatment) and Dex treated animals (Fig 5).

DAVID analysis was used to examine enriched KEGG pathways, which are summarized in S4 and S5 Tables. At 3 h post-IR, all groups (Vehicle, Dex, VPA) demonstrated enriched MAPK (mitogen-activated protein kinases) signaling, consistent with an ischemic stress response. While animals with Vehicle and Dex showed 29 and 30 members of MAPK signaling pathway upregulated, respectively, those animals treated with VPA showed only16 members upregulated, indicating a decreased VPA-induced stress response. At 24 h, DNA replication and cell cycle pathways were upregulated in all groups. However, animals treated with VPA had a larger number of DNA replication and cell cycle pathway related genes upregulated at 24 h (S4 Table) compared to Vehicle or Dex treatment. Mediators of ECM (extracellular matrix)-receptor interactions were not apparent at 24 h in Vehicle and Dex treated animals, but were evident in VPA treated animals; however, at 120 h, these mediators were upregulated in Vehicle and Dex treated groups, but not in VPA treated animals.

Pathophysiological events occurring in the injured kidney are captured in the phenotypic changes found in the gene expression profiles [29, 30]. In our recent study, we found significant correlation of up and down regulated KEGG pathways in both gene and protein data [21]. Not all changes at the gene level, however, constitute changes at the protein level. For example, our microarray data support a large increase at the gene level of KIM-1, analysis at the protein level did not demonstrate an increase at 3h, likely due to the temporal differences in transcription and translation. Further, microarray analysis fails to predict protein activity, post-transcriptional or post-translational modification. Unlike whole genome studies, mass spectrometry is even more difficult because the technology allows for only a fraction of the proteome to be analyzed. Reliable measurement of protein abundance with mass spectrometry is also difficult, and ultimately does not provide a quantitative assessment of functional activity.

We decided to query our gene lists with TFactS (www.TFactS.org), a catalog that contains 6401 regulations linking 343 distinct transcription factors (TFs) to 2720 distinct genes[28] in which all TF target genes are considered independently from experimental conditions. The TFactS tool relies on heavily conserved gene regulation in mammals, and combines data from human, mouse and rat, which is justified by the fact that many published experiments were performed in heterogeneous biological systems. Across all time points, alterations in gene transcriptions predicted 16 regulated TFs in animals with no treatment (7 at 3 h, 1 at 24 h, and 8 at 120 h), 11 in animals treated with Dex (4 at 3 h, 5 at 24 h, and 2 at 120 h), and 13 in animals treated with VPA (11 at 3 h and 2 at 24 h) for a total of 245 TF-gene relationships (Table 4). Compilation of the full analysis is available in S6 Table. Interestingly, all of the 245 predicted up and down regulated genes based on the threshold values we used (<2% RC, P<0.01, FDR<0.01, and > 2 TF-gene relationships) were corroborated with actual gene microarray expression data (see S3 Table for complete DEG analysis), suggesting the TFactS tool is appropriate to identify putative pathway targets.

**Table 4:** Regulated transcription factors and their predicted genes that regulated up or down.

TF	P	FDR	I	TG	RC%	Tx	H	Predicted Genes Regulated
JUND	0.00E+00	2.89E-03	9	28	0	None	3	Up-JUN,F3,PLAUR,VGF,IL6,TIMP1,NR4A1,CDKN1A,FOSL1
ATF1	0.00E+00	6.61E-03	9	59	1	None	3	Up-SLC20A1,JUN,HK2,IL6,HSPA5,FOS,PLAUR,INHBA; Down-UGT8
STAT1	2.00E-05	8.26E-03	8	61	1	None	3	Up-ICAM1,CSF1,IRF1,FOS,CXCL10,SOCS3,TIMP1,CDKN1A
HIF1A	2.00E-05	8.68E-03	7	45	0	None	3	Up-PFKFB3,ADM,HMOX1,NR4A1,CTGF,SERPINE1,TIMP1
JUNB	0.00E+00	3.31E-03	7	20	1	None	3	Up-TGM1,VGF,CDKN1A,IL6,PLAUR,TIMP1,JUN
PPARG	1.00E-05	7.44E-03	7	41	1	None	3	Up-SERPINE1,NR1D1,SAT1,GADD45A,GADD45B,CDNK1A,ANGPTL4
PPARD	1.30E-04	9.92E-03	4	14	1	None	3	Up-SERPINE1,GADD45B,ANGPTL4,GADD45A
ETV4	1.40E-04	8.88E-03	7	31	1	None	24	Up-TIMP1,VIM,MMP3,PLAUR,PCNA,RAD51; Down-SLC9A3
SMAD3	0.00E+00	9.52E-04	9	63	1	None	120	Up-TAGLN,COL6A3,TIMP1,VIM,COL6A1,COL6A2,FN1,COL1A1; Down-TNFSF11
NOTCH1	1.00E-05	1.91E-03	6	40	0	None	120	Up-MCM6,MYC,TAGLN,ADAM19; Down-TNFRSF11,REN
TCF7L2	7.00E-05	4.29E-03	6	61	1	None	120	Up-CD44,TAGLN,VIM,MYC,MYBL2; Down-TNFRSF11
ETV4	4.10E-04	6.67E-03	4	31	1	None	120	Up-TIMP1,VIM; Down-SLC9A3,PTGS2
SMAD7	6.10E-04	7.62E-03	3	15	0	None	120	Up-COL6A2,COL6A3,COL1A1
SMAD4	1.73E-03	8.10E-03	4	45	0	None	120	Up-TIMP1,TAGLN,CLDN1; Down-TNFRSF11
NFIC	1.73E-03	8.57E-03	4	45	0	None	120	Up-CLU,COL1A1; Down-SLC2A4,SLC9A3
SMAD1	1.96E-03	9.05E-03	3	22	0	None	120	Up-TAGLN,MYC; Down-TNFRSF11
SMAD3	0.00E+00	3.72E-03	10	63	1	Dex	3	Up-CTGF,VIM,SERPINE1,EDN1,ZFP36,BAMBI,JUN,TIMP1,PTHLH,CYR61
JUNB	0.00E+00	6.61E-03	7	20	1	Dex	3	Up-IL6,TIMP1,CDKN1A,PLAUR,VGF,TGM1,JUN
ATF4	3.00E-05	8.68E-03	5	18	1	Dex	3	Up-HSPA5,HMOX1,DDIT3,PPP1R15A,ASNS
HIF1A	4.00E-05	9.09E-03	7	45	1	Dex	3	Up-SERPINE1,CTGF,PFKB3,HMOX1,TIMP1,NR4A1,ADM
FOSL1	3.00E-05	4.88E-03	3	4	0	Dex	24	Up-CCNA2,PLAUR,FOSL1
NFE2L2	3.00E-05	5.29E-03	5	19	0	Dex	24	Up-NQO1,GPX2,HMOX1; Down-NQO2,GCLC
E2F2	4.00E-05	6.10E-03	6	32	1	Dex	24	Up-MCM6,MYC,CDC6,CDC45,E2F1,MCM5
SMAD1	7.00E-05	6.50E-03	5	22	1	Dex	24	Up-GADD45B,MYC,CDKN1A; Down-TNFRSF11,PDGFC
SMAD4	2.80E-04	9.76E-03	6	45	1	Dex	24	Up-CLDN1,CDKN1A,TIMP1,COL7A1,COL6A2; Down-TNFRSF11,HNF4A
SMAD7	0.00E+00	4.31E-03	7	15	0	Dex	120	Up-COL3A1,COL1A1,COL6A3,HMOX1,COL5A2,COL1A2,COL6A2
SMAD2	3.30E-04	8.94E-03	5	21	1	Dex	120	Up-COL7A1,TGFB1,TAGLN; Down-TNFRSF11,TIMP3
JUN	0.00E+00	5.05E-04	11	131	1	VPA	3	Up-EDN1,SLC20A1,PTX3,PLAUR,DDIT3,TFPI2,JUN,FOSL1,HBEGF,CSF1,IL6
ESR1	0.00E+00	1.01E-03	7	54	0	VPA	3	Up-CDKN1A,MYC,JUN,GADD45B,JUNB,HSPB1,SERPINE1
EGR1	0.00E+00	3.54E-03	9	91	0	VPA	3	Up-IL6,ICAM1,HMOX1,CSF1,GDF15,TFPI2,JUN,CEBPB,SERPINE1
JUND	0.00E+00	4.04E-03	5	28	0	VPA	3	Up-JUN,IL6,CDKN1A,FOSL1,PLAUR
ATF4	1.00E-05	5.56E-03	4	18	0	VPA	3	Up-DDIT3,PPP1R15A,HMOX1,HSPA5
RELA	1.00E-05	6.57E-03	7	84	0	VPA	3	Up-ICAM,GADD45B,CXCL10,MYC,IL6,PTX3,CXCL1
JUNB	2.00E-05	7.07E-03	4	20	0	VPA	3	Up-CDKN1A,PLAUR,IL6,JUN
REL	3.00E-05	8.08E-03	4	23	0	VPA	3	Up-ICAM1,IL6,CXCL1; Down-TNFSF10
SMAD1	3.00E-05	8.59E-03	4	22	0	VPA	3	Up-CDKN1A,GADD45B,BAMBI,MYC
TCF7L2	1.40E-04	9.09E-03	5	61	1	VPA	3	Up-EDN1,MYC,MAP3K6,BAMBI; Down-DKK1
STAT1	1.40E-04	9.60E-03	5	61	0	VPA	3	Up-CDKN1A,CXCL10,CSF1,ICAM1,SOCS3
E2F3	1.00E-05	5.45E-03	9	36	1	VPA	24	Up-MCM5,CDC45,CDKN1A,PLK4,MCM6,CDC6,PLK1,E2F1; Down-PCSK6
E2F2	3.00E-05	7.37E-03	8	32	1	VPA	24	Up-E2F1,CDC6,MCM6,MCM5,PLK1,PLK4,CDC45; Down-PCSK6

TF, Transcription factor; P, Probability; FDR, False Discovery Rate; I, Interaction; TG, Target Gene; RC, Random Control; Tx, Treatment; H, Hours Post-ischemia reperfusion; None, No treatment; Dex, Dexamethazone; VPA, Valproic Acid. All treatment groups (None, Dex, and VPA) were queried with TFactS at each time point (3, 24, and 120 hours) by using the up and down regulated gene lists presented in Supplementary information **S5 Table**. To reduce the complexity of the data, only TF that has a random control <2%, p<0.01, FDR<0.01 and more than two gene targets were presented (see the full queries in supplementary information **S6 Table**). The total number of target genes for a given transcription factor (i.e. JUNB has 20 target genes) is a property of the TFactS database. The intersection (I) of the target genes (TGs) in the microarray analysis with the transcription factors (TFs) in the database is presented.

## Discussion

In the present study, we analyzed certain significant blood and urine bio-markers of kidney injury, and determined the efficacy of VPA and Dex therapy in renal protection. Also, we identified novel early genes and signaling pathways involved in kidney IR injury and recovery with and without VPA or Dex treatment by DNA microarray analysis.

Two agents, VPA and Dex were studied for their efficacy in reducing renal IR injury. In the beginning, VPA was administered at 300 mg/kg as described previously [17]. Unfortunately, this resulted in unacceptably high rate of cardiac arrest and animal death within ten minutes of administration. This result was probably due to using a different rat strain (Wister) which may be more sensitive to VPA, compared to previous successful studies that used the Sprague-Dawley strain. Differences in the sensitivity of rat strains to renal IR has been previously reported [31], but not to VPA to the best of our knowledge. We then reduced VPA dose to 150 mg/kg body weight and administered intraperitoneally throughout the study without any further episodes of sudden cardiac death.

Plasma biomarkers (creatinine and BUN), and urine bio-markers (albumin, lipocalin-2, KIM-1, and osteopontin) were evaluated with the objective to identify potential early and sensitive markers for the diagnosis of renal damage. Histopathology served as the gold standard for comparison. Blood biomarkers creatinine and BUN were sensitive to detect renal IR injury as early as 3 h and at 24 h, which is in agreement with previous reports [32, 33]. However, the creatinine level is influenced by both renal and non-renal factors, and is not reflective of glomerular filtration rate (GFR); therefore, it is not an optimal marker of kidney injury [8, 10, 34].

The reduced levels of BUN and plasma creatinine observed in Dex and VPA treated animals in the present study agree with previous reports [35, 36] that studied acute kidney injury in septic shock models. However, the traditional biomarkers (BUN and creatinine) are currently considered as less sensitive and not specific for the diagnosis and /or prognosis of acute kidney injury [10, 37].

The urine biomarker albumin was significantly higher at 3 h post-IR compared to normal (base) levels and all other urine biomarkers studied (note there were no other time points examined between 3 h and 24 h in this study). Albumin, a major protein in the blood is primarily produced in the liver. When there are structural changes and damage to the podocytes and glomerular basement membrane, albumin leaks into the urine [10]. Also, albuminuria can result from defects in the cell retrieval pathway in the proximal tubules [38]. Urine albumin levels are increased in both glomerular and tubular diseases, but recently it was reported that the gene expression of albumin is increased in acute kidney injury, thus making it a sensitive marker for acute kidney injury [39]. In the present study, both VPA and Dex treatments administered, prior to IR injury significantly reduced the urine albumin level and kidney histopathologic score (reduced kidney damage) at 3 h post-IR. These findings are in agreement with Van Beneden and co-workers [40] who studied the effects of VPA in Adriamycin-induced nephropathy in mice. In their study, there was no proteinuria and glomerulosclerosis development with VPA premedication. Interestingly, they also observed VPA to reverse Adriamycin induced nephropathy when administered following kidney injury. Furthermore, VPA attenuated the renal inflammation, apoptosis, and fibrosis [40].

Urine osteopontin level was not different from base levels at 3 or 24 h post-IR injury, but it was higher at 120 h in the Vehicle control group. So, osteopontin was not an early marker of kidney injury in the present study. In contrast, osteopontin levels were upregulated in some rodent models of drug (gentamycin, cisplatin, cyclosporine etc.) induced nephritis and streptozotocin induced type-I diabetes [41]. However, osteopontin levels were higher in VPA and Dex compared to Vehicle group at 24 h and 120 h post-IR. This finding suggests osteopontin can serve as a marker of kidney injury repair. Osteopontin has a role in bone metabolism, immune regulation, and cell survival [41]. Its upregulation (both mRNA and protein) has also been shown in renal biopsies from patients with tubulointerstitial fibrosis [42, 43]. Therefore, increased osteopontin levels in VPA and Dex treated animals could be interpreted as increased cell turnover or regeneration from damage.

Lipocalin-2 (NGAL) is a protein that is expressed in epithelial cells upon inflammation or malignancy [44]. In the kidney, NGAL is implicated in both progress and protection from renal injury [45, 46]. In a study by Mishra et al. [11], NGAL was upregulated ten-fold in the urine and preceded the rise in serum creatinine in mouse models of renal ischemic injury. Exogenous administration of NGAL has been shown to protect kidney from IR injury by reducing tubular cell apoptosis via inhibiting caspase-3 activation and *Bax* expression [47]. In the present study, urine NGAL levels were higher at 24 h but not at 3 h post-IR similar to the findings by Wunnapuk et al. [48] who observed increased levels of NGAL at 24–48 h, but not earlier, in their herbicide MPCA (4-chloro-2-methylphenoxyacetic acid) induced kidney injury model. Thus, lipocalin-2 was not a very early diagnostic marker of kidney injury. The levels were reduced to base levels by five days in all groups studied and did not appear to be a potential indicator of tissue regeneration and repair. In the present study, administration of VPA and Dex, prior to kidney IR injury did not have any effect on lipocalin-2 levels compared to Vehicle control.

In the present study, urinary KIM-1 levels at 3 h post-IR were not different from base levels but, at 24 h they were significantly higher than at 3 h. Several studies have reported KIM-1 levels to rise within hours of kidney injury [10, 49–52]. Urinary KIM-1 has been shown to be a sensitive marker in both acute and chronic kidney injuries induced by drugs [53], toxins [48], and ischemia [49]. Additionally, human studies have demonstrated urinary KIM-1 as a possible early diagnostic biomarker of acute kidney injury. Han and Co-workers [54] showed increased KIM-1 expression in kidney biopsies, as well as increased levels of urinary KIM-1 in patients with acute tubular necrosis after initial ischemic insult. In the present study, the KIM-1 expression continued to be higher at 120 h post-IR and was probably associated with proliferating and regenerating proximal tubules. Pre-treatment with VPA or Dex had no significant effect on the levels of KIM-1 compared to the Vehicle control group. However, from the present findings KIM-1 and lipocalin-2 appear to be the most promising diagnostic markers of acute kidney injury.

Reduced histopathologic injury in the renal cortex and medulla at 3 h post-IR confirmed the protective effects of VPA against kidney IR injury. These findings concur with previous reports on kidney IR causing acute kidney injury [40] and intestinal IR causing acute lung injury [55]. In the present study, at 24 h post-IR, tubular necrosis was significantly pronounced in the medulla than in the cortex in Vehicle controls. However, VPA administration reduced tubular necrosis markedly in the medulla compared to Vehicle control. Interestingly tubular regeneration was lower in VPA group and higher in Dex group compared to Vehicle controls at 120 h post-IR. To our knowledge, other kidney IR studies have not evaluated the differences in tubular necrosis and regeneration between cortical and medullary tubules to compare our results. Nonetheless, many drug induced kidney toxicity studies have described kidney pathology targeting tubular and glomerular structures: tubular necrosis and regeneration, and glomerulopathy [56].

Significantly lower levels of pro-inflammatory cytokines IL1β and IL6 in the kidneys of VPA treated animals compared to Vehicle observed in this study is in agreement with other IR injury models [55, 57, 58]. Liu and co-workers [58] demonstrated VPA treatment in a rat hemorrhagic shock model to improve survival, suppress myeloperoxidase activity, inhibit pro-inflammatory cytokines TNFα and IL6, and attenuate acute lung injury. Increased BCL2 levels at 3 h and /or 24 h post-IR in VPA and Dex treated animals, indicated probable anti-apoptotic mechanism of action in reducing IR injury and these findings are similar to previous reports [7, 40, 59]. Additionally, the reduction in inflammatory cytokines in conjunction with the decreased histopathologic IR changes further demonstrates the protective effects of VPA and Dex against kidney IR injury.

To identify novel early candidate biomarkers and pathway knowledge associated with IR mediated kidney injury and recovery repair, we performed DNA microarray analysis in animals with no treatment, Dex or VPA compared to naïve rats. Stress induced genes (*Hspa1b* and *Hspb1*) and major regulators of apoptosis (*Atf3*, *Hmox1*, *and Zfand2a*) were upregulated as early as 3 h following IR injury in all groups (VPA, Dex, Vehicle). *Havrc1* (KIM-1), a well-known early biomarker of kidney injury was expressed significantly at 24 h post-IR in all groups. *Timp1*, a well-known biomarker of organ injury, expression was also upregulated at 24 h post-IR which is in agreement with a previous report [40]. In the present study, high levels of *Havcr1* and *Timp1* expression in Vehicle controls even at 120 h post-IR but not in VPA suggested persistent kidney injury without VPA treatment. Gene expressions observed agree with previously reported studies in many models of kidney injury [8, 50, 56]. In animals treated with VPA, stress and apoptosis related gene expressions were far less relative to animals that were untreated (Vehicle controls) or treated with Dex (see pathway analyses below). Our present findings in rat kidney ischemia-reperfusion model (by vascular clamping) further support the previously established roles of VPA in preventing inflammation and apoptosis in ischemia-reperfusion injury [4, 5, 40, 60].

While urine albumin levels were markedly higher at 3 h post-IR but not at 24 or 120 h, there was no evidence at the gene transcription level for *Alb*, which is expected since it was the result of leakage. Our results provide a significant number of candidate genes that could be evaluated early on at the protein level in urine. It is likely, however, that the 3 h time point is too early to detect significant levels of a given protein, despite large changes at the gene transcription level. For example, increased expression of *Havcr* and *Lcn2* (Lipocalin-2) at 3 h in the kidney (microarray data) corresponded with their increased excretion in the urine (biomarker analysis) at 24 h. Therefore, it is not just the gene expression of a molecule by the kidney, but translation and subsequent release or leakage into the urine that is critical. Future studies should focus not only on literature based evidence of early biomarkers, but also computational analysis of gene arrays, high-confidence protein-protein interaction, and some level of experimental qualification, as we have conducted in previous works. [61, 62]

We conducted pathway enrichment because it provides a framework to evaluate the efficacy of the drugs (i.e. level pathway enrichment), identify putative targets for consideration, and provide a basis for inferential analyses in future drug-protein and /or protein-protein interaction studies. This is particularly important since microarray analysis only provides a snapshot in time of changes that are in flux within the kidney. DAVID analysis identified several enriched KEGG pathways. VPA suppressed early MAPK (mitogen-activated protein kinases) signaling with fewer members upregulated (16 members) compared to Vehicle (29 members) and Dex (30 members) treated groups. In all groups most downstream mediators of MAPK signaling were upregulated (*Creb*, *c-Myc*, *Jun*, *JunD*, *Gadd53*, *Hsp27*, *Hsp72*, *MKPs*), whereas only four upstream mediators were upregulated (*Fgf*, *Tnfr*, *Traf6*, *Ask6*). The most pronounced findings in the pathway analyses were that VPA induced expression of a large number of genes related to DNA replication, cell cycle, pyrimidine metabolism, mismatch repair, nucleotide excision, and ECM-receptor interaction very early (at 24 h) compared to other groups. An increase in these pathways may point to a more robust cellular repair and recovery process after ROS (reactive oxygen species)-mediated injury, a known effect related to reperfusion after ischemia [63]. These pathways may enhance recovery from the IR injury, despite continued decrease in mediators of energy and ROS metabolism.

By120 h, post-IR gene expression in VPA treated kidney was indistinguishable from normal kidney (naïve uninjured and untreated) except for two genes that were elevated (*Clec4a2* and *Lox*), neither of which belong to a KEGG network. The *Lox* gene encodes lysyl oxidase that catalyzes the lysine residues to form aldehydes and assists in cross-linking collagen and elastin [64]. *Clec4a2* encodes C-type lectin domain family 4, member a2 (dendritic cell immunoreceptor) that probably regulates immune reactivity [65]. The significance of these two genes being upregulated, however, remains to be determined. Overall, VPA treatment was well-tolerated and markedly reduced IR injury and clearly enhanced recovery in the kidney, as evidence of the pathophysiological phenotype reverting back to the naïve rat profile.

To gain additional insight into the regulated pathways, we performed a transcription factor analysis using a web-based tool called TFactS (www.TFactS.org). To use this tool, we input the up and down regulated genes from our microarray into the query to predict whether transcription factors were regulated in the pathophysiological state of the kidney with no treatment, Dex, or VPA. While the primary aim of this tool is to interpret human data, the tool also supports rat and human orthologues. Using the Sign-Sensitive catalogue (which incorporates up and down regulation information), we identified a large number of potentially regulated TFs, which we reduced in complexity by applying several threshold, as described in the methods. Interestingly, this reduced the number of transcription factor-target gene (TF-TG) interactions to 245, of which all were confirmed by actual expression data in the microarray analysis, suggesting that TFactS could be used to mine putative gene targets for therapeutic approach.

In our model of ischemia-reperfusion injury, TF analysis provided a significant information as to the therapeutic value of our treatments (VPA or Dex) used. Several observations can be made from the Transcription analysis data (Table 4). Transcription factor Hypoxia inducible factor-A (HIFA) was not observed in VPA treated kidney at 3 h post-IR but was present in untreated control suggesting injury response to low oxygen concentration was more pronounced in the control and dampened/no response in the VPA treated animal. Another example would be JUNB-TIMP1 (TF-TG) interaction was not observed in VPA treated group compared to untreated controls at 3 h post-IR. TIMP1 is a tissue inhibitor of metalloproteinases that inhibits degradatation of extracellular matrix, promotes cell proliferation, and has anti-apoptotic activity. However, our analysis showed no association with TIMP1 upregulation via JUNB transcription factor in VPA treated animals.

Overall, our study provided novel candidate biomarkers of IR kidney injury for further analysis, provided strong evidence and support for the use of kidney injury (KIM-1, lipocalin-2, albumin) as early as 24 h in the diagnosis of kidney injury, and provided pathway knowledge associated with VPA-mediated recovery. The VPA therapy seems to be promising to mitigate kidney IR injury, essentially indistinguishable from naive animals at 120 h. Furthermore, we have presented a comprehensive analysis of microarray data on VPA and Dex induced gene expression in the rat ischemic kidney injury model for the first time, which can be used as the basis for further drug-protein interaction and protein-protein interaction studies.

## Supporting Information

S1 Table Top twenty upregulated gene expression in rat kidney ischemia-reperfusion injury with and without treatment. (DOCX)

S2 Table Top twenty downregulated gene expression in rat kidney ischemia-reperfusion injury with and without treatment. (DOCX)

S3 Table Complete list of Differentially Expressed Genes (DEGs). (XLSX)

S4 Table Upregulated KEGG (Kyoto encyclopedia of genes and genomes) Pathways showing possible molecular interactions in rat kidney ischemia-reperfusion (IR) injury with and without treatment. (DOCX)

S5 Table Downregulated KEGG (Kyoto Encyclopedia of genes and genomes) Pathways showing possible molecular interactions in rat kidney ischemia-reperfusion (IR) injury with and without treatment. (DOCX)

S6 Table Complete TFactS Analysis. (XLSX)

S1 Text Animal Research: Reporting *In Vivo* Experiments (ARRIVE). (DOCX)
