# Supplementary material for: Effects of Valproic Acid and Dexamethasone Administration on Early Bio-Markers and Gene Expression Profile in Acute Kidney Ischemia-Reperfusion Injury in the Rat
Source: PLoS One. 2015 May 13;10(5):e0126622. doi: 10.1371/journal.pone.0126622 (PMC4430309; doi:10.1371/journal.pone.0126622)
Supplement: S1 Table — (DOCX) [file pone.0126622.s001.docx]

| **No Treatment (Vehicle)** | | | | | | **Dexamethasone (Dex) Treatment** | | | | | | **Valproic Acid (VPA) Treatment** | | | | | |
| --- | --- | --- | --- | --- | --- | --- | --- | --- | --- | --- | --- | --- | --- | --- | --- | --- | --- |
| **3 hours** | | **24 hours** | | **120 hours** | | **3 hours** | | **24 hours** | | **120 hours** | | **3 hours** | | **24 hours** | | **120 hours**** | |
| **Gene** | **Fold** | **Gene** | **Fold** | **Gene** | **Fold** | **Gene** | **Fold** | **Gene** | **Fold** | **Gene** | **Fold** | **Gene** | **Fold** | **Gene** | **Fold** | **Gene** | **Fold** |
| Hspa1b | 23.3 | Havcr1 | 47.1 | Havcr1 | 28.8 | Atf3 | 32.0 | Havcr1 | 56.2 | Havcr1 | 76.2 | Hspa1b | 16.8 | Havcr1 | 87.4 | Lox | 3.6 |
| Hspa1b | 22.9 | Fosl1 | 14.7 | Lcn2 | 10.5 | Hspa1b | 24.8 | Lcn2 | 16.2 | Lcn2 | 19.5 | Hmox1 | 14.2 | Lcn2 | 16.4 | Clec4a2 | 2.9 |
| Atf3 | 22.4 | Lamc2 | 11.8 | Fgb | 7.0 | Hspa1b | 19.8 | Gal | 8.3 | Il19 | 16.2 | Atf3 | 10.8 | Fgb | 14.1 |  |  |
| Hmox1 | 21.0 | Lcn2 | 10.7 | A2m | 6.4 | Hmox1 | 16.9 | Rdm1 | 7.9 | Fgb | 11.8 | Hspa1b | 10.2 | Tgm1 | 11.9 |  |  |
| Fgf23 | 16.2 | Hmox1 | 10.3 | Il19 | 5.4 | Fosl1 | 16.6 | Ptk6 | 6.6 | Il24 | 11.1 | Zfand2a | 9.6 | Lamc2 | 11.5 |  |  |
| Fosl1 | 14.1 | Tgm1 | 10.2 | Fam129a | 5.2 | Zfand2a | 15.1 | Fosl1 | 6.6 | A2m | 10.3 | Fosl1 | 7.7 | Il19 | 10.3 |  |  |
| Zfand2a | 12.4 | Timp1 | 9.8 | Ptk6 | 4.8 | Hbegf | 13.2 | Tgm1 | 6.5 | Gpnmb | 9.3 | Hspb1 | 6.6 | Timp1 | 9.9 |  |  |
| Rnd1 | 12.1 | Hspa1b | 8.9 | Adra1d | 4.7 | Apold1 | 13.2 | Timp1 | 6.0 | Adra1d | 9.3 | Ptk6 | 6.4 | Fosl1 | 9.7 |  |  |
| Hspb1 | 11.5 | Olr1 | 7.8 | Il24 | 4.7 | Havcr1 | 11.9 |  | 5.5 | Fam129a | 7.8 | Chac1 | 6.1 | Cyp3a9 | 8.2 |  |  |
| Hbegf | 10.2 | Rnd1 | 7.4 | Gpnmb | 4.6 | Gadd45b | 11.7 | A2m | 5.5 | Clu | 7.4 | Tnfrsf12a | 5.8 | Il24 | 8.2 |  |  |
| Selp | 9.7 | Tubb6 | 7.3 | B4galnt4 | 4.6 | Hspb1 | 11.5 | Glycam1 | 5.5 | Cthrc1 | 7.4 | Rnd1 | 5.5 | Ucma | 8.1 |  |  |
| Socs3 | 8.7 | PVR | 7.1 | Timp1 | 4.4 | Nppc | 10.8 | Fgb | 5.1 | Cyp24a1 | 7.3 | Socs3 | 5.4 | Tubb6 | 8.1 |  |  |
| Prss22 | 8.2 | Socs3 | 7.1 | Cthrc1 | 4.3 | LOC100360880 | 10.4 | Lamc2 | 5.0 | Ptk6 | 7.3 | Srxn1 | 5.3 | Clu | 8.0 |  |  |
| Chac1 | 7.8 | Atf3 | 7.0 | Lox | 4.3 | Rnd1 | 10.0 | Angptl4 | 4.7 | Cd44 | 7.0 | Dnajb1 | 5.0 | Ptk6 | 7.8 |  |  |
| Rgs16 | 7.8 | Il19 | 6.9 | Cd44 | 4.3 | Chac1 | 9.7 | Dtl | 4.6 | Slc34a2 | 6.8 | Hbegf | 4.9 | Lamb3 | 7.4 |  |  |
| -- | 7.4 | Krt17 | 6.7 | Col8a1 | 4.2 | Klf6 | 9.5 | Lox | 4.6 | Trpv6 | 6.8 | Myc | 4.8 | Olr1 | 7.3 |  |  |
| Havcr1 | 7.4 | Gabrp | 6.7 | Tubb6 | 4.0 | Gal | 9.5 | Serpina3n | 4.5 | Col8a1 | 6.3 | Ddit3 | 4.6 | B4galnt4 | 7.1 |  |  |
| Plaur | 7.2 | Myc | 6.6 | Gabrp | 4.0 | Myc | 9.3 | Olr1 | 4.5 | Timp1 | 6.3 | PVR | 4.5 | Adra1d | 6.9 |  |  |
| Dnajb1 | 7.1 | Tnfrsf12a | 6.6 | Cyp24a1 | 4.0 | Prss22 | 9.1 | Mcm6 | 4.4 | Adamts1 | 6.0 | Angptl4 | 4.3 | Gabrp | 6.8 |  |  |
| Klf6 | 7.0 | Zfand2a | 6.6 | Slc34a2 | 4.0 | Srxn1 | 9.0 |  | 4.3 | Tubb6 | 5.9 | Hsph1 | 4.2 | Mcm6 | 6.7 |  |  |

**S1 Table. Top twenty upregulated gene expression in rat kidney ischemia-reperfusion injury with and without treatment***

*, Gene expression levels were compared with normal (naïve) uninjured and untreated animals; **, At 120 hours only two genes were upregulated in

VPA treated group; --, Unknown gene
