# Supplementary material for: Effects of Valproic Acid and Dexamethasone Administration on Early Bio-Markers and Gene Expression Profile in Acute Kidney Ischemia-Reperfusion Injury in the Rat
Source: PLoS One. 2015 May 13;10(5):e0126622. doi: 10.1371/journal.pone.0126622 (PMC4430309; doi:10.1371/journal.pone.0126622)
Supplement: S2 Table — (DOCX) [file pone.0126622.s002.docx]

**S2 Table. Top twenty downregulated gene expression in rat kidney ischemia-reperfusion injury with and without treatment***

| **No Treatment (Vehicle)** | | | | | | **Dexamethasone (Dex) Treatment** | | | | | | **Valproic Acid (VPA) Treatment** | | | | | |
| --- | --- | --- | --- | --- | --- | --- | --- | --- | --- | --- | --- | --- | --- | --- | --- | --- | --- |
| **3 hours** | | **24 hours** | | **120 hours** | | **3 hours** | | **24 hours** | | **120 hours** | | **3 hours** | | **24 hours** | | **120 hours**** | |
| **Gene** | **Fold** | **Gene** | **Fold** | **Gene** | **Fold** | **Gene** | **Fold** | **Gene** | **Fold** | **Gene** | **Fold** | **Gene** | **Fold** | **Gene** | **Fold** | **G** | **F** |
| Dkk1 | -2.3 | -- | -6.6 | Cml1 | -4.8 | Plxnd1 | -2.5 |  | -5.5 | Ppp1r1a | -6.0 |  | -2.2 | Spata22 | -6.2 |  |  |
| Qrsl1 | -2.3 | Slc6a12 | -6.7 | Slc22a9 | -5.0 | Mtmr7 | -2.5 | Slc15a2 | -5.6 | Egf | -6.4 | LOC685849 | -2.3 | Inmt | -6.3 |  |  |
| RGD1565168 | -2.3 | Dnase1 | -6.8 | Anxa13 | -5.1 | Kank4 | -2.5 |  | -6.0 | Dnase1 | -6.4 | LOC686809 | -2.3 | Dhrs7 | -6.5 |  |  |
| Rassf6 | -2.3 | Slc21a4 | -7.1 | Hnmt | -5.1 | Lipg | -2.5 | Slc7a13 | -6.0 | Cyp1a1 | -6.5 | Cnr1 | -2.3 | Slc6a12 | -6.8 |  |  |
| Hmx2 | -2.3 | LOC100360091 | -7.3 | Slco1a6 | -5.4 | Klra1 | -2.5 | LOC689876 | -6.1 | Hnmt | -6.5 | Dkk1 | -2.3 | Slc22a22 | -7.1 |  |  |
| Mir30e | -2.3 | Slco4c1 | -7.3 | Slc6a12 | -5.7 | Dkk1 | -2.6 | Slco4c1 | -6.3 | Tff3 | -7.2 | Lrrc61 | -2.3 | Hnmt | -7.3 |  |  |
| Ppp1r1b | -2.4 | Ppp1r1a | -7.3 | Cyp1a1 | -5.9 |  | -2.6 |  | -6.4 | Slco1a6 | -7.7 | Alx1 | -2.3 | Cyp2c11 | -7.4 |  |  |
|  | -2.4 | Slc22a22 | -7.4 | RGD1564347 | -6.1 | LOC686809 | -2.6 | Inmt | -6.6 | Spata22 | -8.1 | Ccno | -2.4 | Cml1 | -7.5 |  |  |
| Mir455 | -2.4 | Dhrs7 | -7.5 | Spata22 | -6.1 | Sox18 | -2.7 | Slco1a6 | -6.9 | Slc6a12 | -9.3 | Ly86 | -2.4 | Slc22a9 | -7.6 |  |  |
| -- | -2.5 | -- | -7.8 | Tff3 | -6.1 | Gp2 | -2.7 | Pamr1 | -7.1 | Gucy1b2 | -10.4 | Tnfsf10 | -2.4 |  | -7.6 |  |  |
| -- | -2.6 | Slc15a2 | -7.9 | Idi2l | -6.1 | Cd180 | -2.7 | Cml1 | -7.4 | Rgn | -11.4 | Cd200 | -2.5 | Gucy1b2 | -7.8 |  |  |
| -- | -2.7 | Hnmt | -9.1 | Gucy1b2 | -6.1 |  | -2.8 | Spata22 | -7.7 | RGD1564347 | -11.5 | Rilp | -2.5 |  | -7.9 |  |  |
| Arhgap4 | -2.7 | Slco1a6 | -9.2 | Dhrs7 | -6.9 | Hmx2 | -2.9 | Slco1a5 | -7.9 | Idi2l | -11.7 | Cmah | -2.5 | Slco1a6 | -8.0 |  |  |
| Agbl3 | -2.9 | Slc22a9 | -9.4 | Rgn | -7.7 | Cmah | -3.0 |  | -8.3 | Cyp2c11 | -12.1 | Adora3 | -2.5 | Egf | -9.8 |  |  |
| Lrrc66 | -3.2 | Egf | -9.5 | Cyp2c11 | -7.8 |  | -3.2 | Gucy1b2 | -8.4 |  | -12.5 | Gucy1a3 | -2.7 | Dnase1 | -10.0 |  |  |
| Tril | -3.2 | Slco1a5 | -9.6 | -- | -8.5 | Cx3cr1 | -3.3 | Cacng5 | -8.6 | Dhrs7 | -12.7 | Casr | -2.7 | Cacng5 | -10.1 |  |  |
| Gp2 | -3.2 | Rgn | -10.6 | Slco1a5 | -9.9 | Ugt8 | -3.6 | Hnmt | -9.1 | Slco1a5 | -21.6 | Slco2b1 | -2.7 | Slco1a5 | -10.2 |  |  |
| Mtmr7 | -3.3 | LOC361914 | -10.7 | Cacng5 | -13.2 | Aplnr | -4.2 | Slc7a12 | -10.1 | Cacng5 | -23.6 | Aplnr | -2.7 | Rgn | -10.2 |  |  |
| Ugt8 | -3.3 | Cacng5 | -11.1 | Slc7a12 | -21.3 | Tril | -4.3 | Rgn | -11.0 | Slc7a12 | -35.2 | Cd180 | -2.8 | Slc7a12 | -11.5 |  |  |
| Casr | -3.5 | Slc7a12 | -11.5 | LOC361914 | -25.3 | Casr | -4.3 | LOC361914 | -11.1 | LOC361914 | -37.5 | Tril | -4.0 | LOC361914 | -11.6 |  |  |

*, Gene expression levels were compared with normal (naïve) uninjured and untreated animals; **, At 120 hours no genes were downregulated in VPA

treated group; --, Unknown gene; -, Downregulation
