# Supplementary material for: Effects of Valproic Acid and Dexamethasone Administration on Early Bio-Markers and Gene Expression Profile in Acute Kidney Ischemia-Reperfusion Injury in the Rat
Source: PLoS One. 2015 May 13;10(5):e0126622. doi: 10.1371/journal.pone.0126622 (PMC4430309; doi:10.1371/journal.pone.0126622)
Supplement: S4 Table — (DOCX) [file pone.0126622.s004.docx]

**S4 Table. Upregulated KEGG (Kyoto encyclopedia of genes and genomes) Pathways showing possible molecular interactions in**

**rat kidney ischemia-reperfusion (IR) injury with and without treatment ***

|  |  | No treatment (Vehicle) | | | Dexamethasone (Dex) | | | Valproic Acid (VPA) | | |
| --- | --- | --- | --- | --- | --- | --- | --- | --- | --- | --- |
| Post-IR | Term | Gene  count | % | Fold Enriched | Gene count | % | Fold Enriched | Gene count | % | Fold Enriched |
| 3 hours | rno04010:MAPK signaling pathway | 29 | 7.3 | 4.5 | 30 | 5.7 | 4.1 | 16 | 8.8 | 5.9 |
|  | rno04640:Hematopoietic cell lineage | 11 | 2.8 | 5.8 | - | - | - | - | - | - |
| 24 hours | rno03030:DNA replication | 13 | 2.1 | 10.0 | 14 | 4.2 | 18.6 | 38 | 4.8 | 6.4 |
|  | rno04110:Cell cycle | 22 | 3.5 | 4.7 | 21 | 6.3 | 7.8 | 21 | 2.7 | 12.7 |
|  | rno05322:Systemic lupus erythematosus | - | - | - | 12 | 3.6 | 6.2 | 19 | 2.4 | 4.5 |
|  | rno00240:Pyramidine metabolism | - | - | - | 12 | 3.6 | 6.1 | 19 | 2.4 | 4.4 |
|  | mo03430:Mismatch repair | - | - | - | - | - | - | 9 | 1.1 | 8.6 |
|  | rno04512:ECM-receptor interaction | - | - | - | - | - | - | 16 | 2.0 | 4.2 |
|  | Rno03420:Nucleotide excision repair | - | - | - | - | - | - | 11 | 1.4 | 5.4 |
| 120 hours | rno04512:ECM-receptor interaction | 11 | 5.0 | 10.0 | 20 | 3.1 | 6.6 | - | - | - |
|  | rno04510:Focal adhesion | 13 | 5.9 | 4.9 | 25 | 3.9 | 3.4 | - | - | - |
|  | rno05322-Systemic lupus erythematosus | 9 | 4.1 | 7.4 | 19 | 3.0 | 5.6 | - | - | - |
|  | rno04110:Cell cycle | - | - | - | 22 | 3.4 | 4.6 | - | - | - |

*, Gene enrichment was significant at P≤ 0.001; %, per cent of total genes upregulated; -, none detected
