# Supplementary material for: Effects of Valproic Acid and Dexamethasone Administration on Early Bio-Markers and Gene Expression Profile in Acute Kidney Ischemia-Reperfusion Injury in the Rat
Source: PLoS One. 2015 May 13;10(5):e0126622. doi: 10.1371/journal.pone.0126622 (PMC4430309; doi:10.1371/journal.pone.0126622)
Supplement: S5 Table — (DOCX) [file pone.0126622.s005.docx]

**S5 Table. Downregulated KEGG (Kyoto Encyclopedia of genes and genomes) Pathways showing possible molecular interactions**

**in rat kidney ischemia-reperfusion (IR) injury with and without treatment ***

|  |  | No treatment (Vehicle) | | | Dexamethasone (Dex) | | | Valproic Acid (VPA) | | |
| --- | --- | --- | --- | --- | --- | --- | --- | --- | --- | --- |
| Post-IR | Term | Gene  count | % | Fold Enriched | Gene count | % | Fold Enriched | Gene count | % | Fold Enriched |
| 24 hours | Rno00280:Valine, leucine and isoleucine degradation | 16 | 2.7 | 8.9 | 9 | 2.5 | 8.7 | 18 | 3.6 | 11.6 |
|  | Rno00380:Tryptophan metabolism | 15 | 2.5 | 8.9 | 10 | 2.8 | 10.4 | 16 | 3.2 | 11.1 |
|  | Rno00640:Propanoate metabolism | 12 | 2.0 | 9.3 | - | - | - | 12 | 2.4 | 10.8 |
|  | Rno00982:Drug metabolism | 16 | 2.7 | 5.7 | - | - | - | 16 | 3.2 | 6.6 |
|  | Rno00480:Glutathione metabolism | 13 | 2.2 | 6.6 | - | - | - | - | - | - |
|  | Rno00410:beta-Alanine memtabolism | 9 | 1.5 | 10.4 | - | - | - | 10 | 2.0 | 13.5 |
|  | Rno00620:Pyruvate metabolism | 10 | 1.7 | 6.5 | - | - | - | 9 | 1.8 | 6.9 |
|  | Rno00260:Glycine, serine, and thereonine metabolism | 9 | 1.5 | 7.2 | - | - | - | 9 | 1.8 | 8.1 |
|  | Rno00650:Butonoate metabolism | 9 | 1.5 | 7.0 | - | - | - | - | - | - |
|  | Rno04512:ECM-receptor interaction | - | - | - | 10 | 3.0 | 5.8 | - | - | - |
|  | Rno00340:Histidine metabolism | - | - | - | - | - | - | 8 | 1.6 | 9.9 |
| 120 hours | Rno00982:Drug metabolism | - | - | - | 11 | 2.6 | 6.7 | - | - | - |

*, Gene enrichment was significant at P≤ 0.001; %, per cent of total genes downregulated; -, none detected
