# Supplementary material for: Effects of Valproic Acid and Dexamethasone Administration on Early Bio-Markers and Gene Expression Profile in Acute Kidney Ischemia-Reperfusion Injury in the Rat
Source: PLoS One. 2015 May 13;10(5):e0126622. doi: 10.1371/journal.pone.0126622 (PMC4430309; doi:10.1371/journal.pone.0126622)
Supplement: S1 Text — (DOCX) [file pone.0126622.s007.docx]

**THE ARRIVE GUIDELINES**

**Animal Research: Reporting *In Vivo* Experiments**

**1. Title**

Renoprotective Effects of Valproic Acid and Dexamethasone in Acute Kidney Ischemia-Reperfusion Injury

**2. Abstract**

BACKGROUND AND PURPOSE: Renal ischemia-reperfusion (IR) causes acute kidney injury (AKI) with high mortality and morbidity. The objective of this investigation was to ameliorate kidney IR injury and identify novel biomarkers for kidney injury and repair.

EXPERIMENTAL APPROACH: Under general anesthesia left renal ischemia was induced in Wister rats by occluding renal artery for 45 minutes, followed by reperfusion and right nephrectomy. Thirty minutes prior to ischemia, rats (n=8/group) received Valproic Acid (150 mg/kg; VPA), Dexamethasone (3 mg/kg; Dex) or Vehicle (saline) intraperitoneally. Animals were sacrificed humanely at 3, 24 or 120 h post-IR and tissue (blood, kidney) and urine samples were harvested for biochemical and cellular analysis.

KEY RESULTS: Plasma creatinine (mg/dL) at 24 h was reduced (P<0.05) in VPA (2.7±1.8) and Dex (2.3±1.2) compared to Vehicle (3.8±0.5) group. At 3 h, urine albumin (mg/mL) was higher in Vehicle (1.47±0.10), VPA (0.84±0.62) and Dex (1.04±0.73) compared to naïve (uninjured/untreated control) (0.14±0.26) group. At 24 h post-IR urine lipocalin-2 (µg/mL) was higher (P<0.05) in VPA, Dex and Vehicle groups (9.61-11.36) compared to naïve group (0.67±0.29); also, kidney injury molecule-1 (KIM-1; ng/mL) was higher (P<0.05) in VPA, Dex and Vehicle groups (13.7-18.7) compared to naïve group (1.7±1.9). Histopathology demonstrated reduced (P<0.05) ischemic injury in the renal cortex in VPA (Grade 1.6±1.5) compared to Vehicle (Grade 2.9±1.1). Inflammatory cytokines IL1β and IL6 were downregulated and anti-apoptotic molecule BCL2 was upregulated in VPA group. Furthermore, kidney DNA microarray demonstrated reduced injury, stress, and apoptosis related gene expression in the VPA administered rats.

CONCLUSIONS AND IMPLICATIONS: VPA appears to ameliorate kidney IR injury via reduced inflammatory cytokine, apoptosis/stress related gene expression, and improved regeneration. KIM-1, lipocalin-2 and albumin appear to be promising early urine biomarkers for the diagnosis of AKI.

**3. BACKGROUND**

The kidney is exquisitely sensitive to variations in blood flow [[1](#_ENREF_1)] . Reduced or complete stoppage of blood flow to the kidney during perioperative conditions to prevent bleeding can lead to kidney damage and dysfunction. The risk for long-term renal dysfunction is significantly increased when there is underlying chronic kidney disease (CKD). Globally, the CKD is increasing as diabetes and hypertension continue to rise. In the US, the CKD prevalence has increased from 10% in 1994 to 13% in 2004 [[2](#_ENREF_2)]. Approximately 65,150 new diagnoses of Renal Cell Carcinoma (RCC) were made in the year 2013 in the US [[3](#_ENREF_3)]. Based on Seer Cancer Statistics review 1975-2011, RCC is more commonly seen in older population, with a mean age at diagnosis of 64 years [[4](#_ENREF_4)]. Partial nephrectomy, or nephron-sparing surgery, has been described since 1890 although widespread use of this technique has occurred only over the past 20 years [[5](#_ENREF_5)]. Current indications for partial nephrectomy include conditions that result in patients becoming anephric or at high risk for dialysis, patients with bilateral synchronous tumors, and patients with solitary kidneys [[6](#_ENREF_6)].

Currently, perioperative renal dysfunction prevention in patients with underlying chronic kidney disease is not well understood. Studies of perioperative renal dysfunction are complicated by the lack of a single endpoint scientifically or clinically proven to define renal dysfunction [[7](#_ENREF_7)]. However, outcome measures commonly used in the evaluation of normal physiology and renal function include serum/plasma creatinine, blood flow to the kidney, sodium excretion and urine output. All of these end points have their benefits, but also several shortcomings.

Valproic acid (VPA), an anti-epileptic agent has been shown to have anti-inflammatory and anti-apoptotic properties in ischemic injury [[8](#_ENREF_8),[9](#_ENREF_9)]. VPA via histone deacetylase inhibition enhances nuclear histone acetylation that increases gene transcription and appears to protect cells from apoptosis through the β catenin survival pathway. The β catenin survival pathway involves serine threonine kinase (Akt), phosphatidylinositide 3-kinase (PI3K) and the interaction of BAD (BCL2-associated death promoter**)** with BCL2 (B-cell lymphoma 2) proteins. It appears phosphor-Akt phosphorylates BAD, a pro-apoptotic protein. The phosphorylation of BAD enables BCL2 to act as an anti-apoptotic factor and promote cell survival [[10](#_ENREF_10)]. Thus, it appears VPA has the potential to lead cells towards a survival pathway. Dexamethasone (Dex) has been shown to ameliorate renal IR injury after 24 hours following a single pre-ischemia dose. The exact mechanism for proximal tubule protection is not known. However, it has been shown to be protective in cardiac and neuronal tissue through a non-genomic pathway, activating endothelial nitric oxide synthase system via the PI3K and Akt pathways [[11](#_ENREF_11)].

Lipocalin-2 also called neutrophil gelatinase-associated lipocalin (NGAL); osteopontin (bone phosphoprotein); KIM-1 (kidney injury molecule 1) also called TIM-1 (T Cell immunoglobulin and mucin domain containing protein-1) and HAVCR (Hepatitis A Virus Cellular Receptor 1), and albumin are some of the novel potential urine biomarkers of acute kidney injury [[12-17](#_ENREF_12)]. However, conclusive evidence on their timeline of appearance and disappearance following renal injury is still lacking. DNA microarray analysis for gene expression (mRNA transcripts) is an interesting approach to make an initial assessment of novel markers that appear and /or disappear with renal IR injury and treatment [[18](#_ENREF_18)].

The need for improved therapeutic measures to mitigate perioperative renal dysfunction and early diagnosis of renal dysfunction encouraged us to perform this study. For this purpose, we selected a well-established rat ischemia-reperfusion injury model. The size of the rat allowed easy surgical manipulation and high survival rates. Furthermore, the kidney physiology in rat has been shown to be comparable to human kidney physiology. Our study included both urine and kidney tissue analyses in the rat kidney IR injury model and provided strong evidence and support for the use of early biomarkers (KIM-1, lipocalin-2, albumin) in the diagnosis of kidney injury. Also, rat IR injury model served well to test the novel drugs VPA and Dexamethasone to prevent ischemia-reperfusion injury this study.

**4. Objectives**

The objectives of this study were: 1) to evaluate the potential benefits of novel agents, VPA and Dexamethasone in renal protection from ischemia-reperfusion injury, and 2) to identify novel early biomarkers of kidney injury and repair.

**5. Ethical Statement**

The care and use of the rats were approved by the animal Experiment Ethics Committee of Madigan Army Medical Center. The protocol (No. 212128) was approved by the Madigan Army Medical Center’s Institutional Animal Care and Use Committee (IACUC) on the Ethics of Animal Experiments. All animal experiments were performed according to institutional guidelines with the prior approval by the IACUC. All surgery was performed under anesthesia, and all efforts were made to minimize the animal suffering.

**6. Study Design**

The study included three experimental groups. Group A, VPA treatment; Group B, Dex treatment; and Group C, No treatment (Vehicle i.e., saline control). Treatments were administered before inducing ischemia. Rats underwent left renal ischemia for 45 min, followed by the removal of renal artery clamp (to allow reperfusion) and right nephrectomy. Following reperfusion, animals were sacrificed at 3, 24 or 120 h (h= hour/s; n=8/group). In the 3 h group, rats were maintained under anesthesia after surgery until sacrifice. In the 24 and 120 h groups, the rats were recovered and returned to the cages for normal housing. Analgesic buprenorphine (0.03 mg/kg) was injected at 12 h intervals for three days post-operatively. After animal sacrifice, urine, blood, and kidney were collected for kidney functional biomarker assays, histology and/or molecular analyses. Kidneys and urine samples collected from normal (naïve) uninjured/untreated animals (n=5) immediately following general anesthesia and sacrifice were used for baseline measurements (Figure 1).


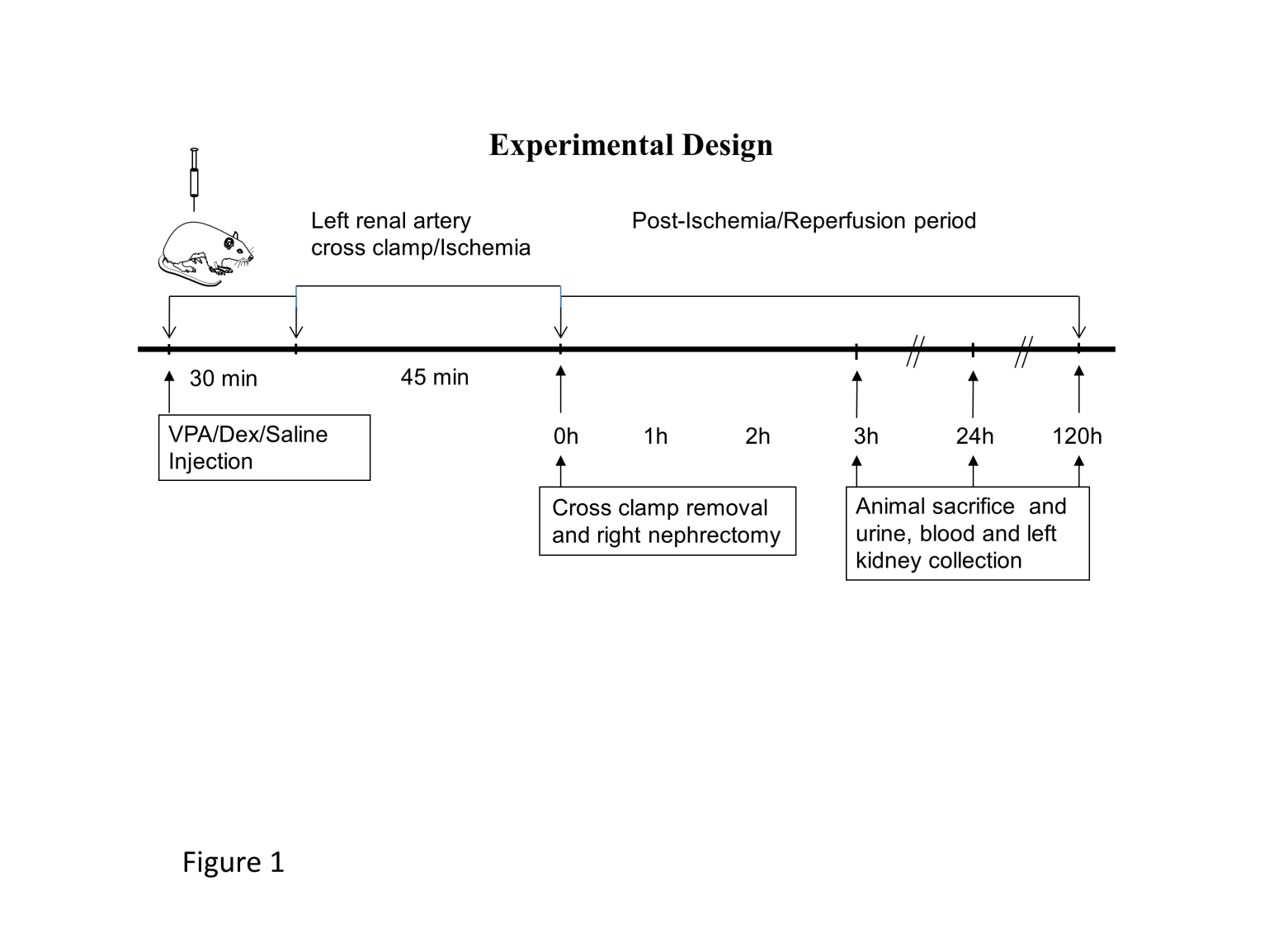


**Figure 1.** **Experimental design.** Lewis rats were pre-medicated with Valproic acid (VPA; 150 mg/kg body weight) or Dexamethasone (Dex; 3 mg/kg/body weight) or saline (Vehicle control) intraperitoneally 30 minutes (min) prior to cross clamping left renal artery and inducing left renal ischemia. The cross clamp was removed after 45 min allowing kidney reperfusion, and at the same time a right nephrectomy was performed. Animals were sacrificed at 3, 24 and 120 hours post ischemia-reperfusion. Left kidney, blood and urine were collected for cellular and molecular analyses.

**7. Experimental Procedures**

Rat renal IR injury models have been used to create proximal tubular damage using warm ischemia [[19](#_ENREF_19)]. Previously published rat kidney IR injury model [[20](#_ENREF_20)] was used, but >80% of the rats died within three days post-IR (n= 9). The procedure was slightly modified by decreasing ischemia time and performing right nephrectomy following reperfusion of the ischemic left kidney allowing rat survival ≥5 days. The model was designed with the objective of creating underlying decreased nephron mass and rat survival of at least five days to assess the perioperative renal dysfunction, as well as recovery. Rats were premedicated with VPA or Dex, prior to ischemia induction in an attempt to protect the renal parenchyma.

Briefly, rats were anesthetized by isoflurane inhalation induction followed by ketamine (~70 mg/kg) and xylazine (~6 mg/kg) administration intraperitoneally. Rats were premedicated with Valproic acid [[21](#_ENREF_21)] at 150 mg/kg, Dex at 3 mg/kg [[11](#_ENREF_11)] or Vehicle (saline control) intraperitoneally thirty minutes prior to surgery. A sterile surgical site was prepared, and a midline incision was performed to access the abdominal cavity. The left kidney was located, and its renal artery was clamped using an atraumatic microaneurysm clamp. Renal ischemia was confirmed by the kidney color which was pale. The abdomen was approximated to prevent dehydration and maintain body temperature. Animal was kept on heat controlled thermal pads to maintain body temperature throughout the procedure. Following 45 min (minutes) of warm ischemia the abdomen was reopened and the renal clamp was removed. Change in the kidney color (pale to bright red) confirmed proper reperfusion. Reperfusion was quickly followed by a right nephrectomy. Finally, the abdomen was closed by suturing muscles and /or fascia with 6-O prolene, and the skin was closed with 4-O nylon and clips.

**8. Experimental Animals**

Wister rats (200-300 g; male) purchased from Harlan Laboratories, Indianapolis, IN, USA. We had three experimental groups and 3 time points (3, 24, and 120 h) in each group: Group A (VPA treatment; n=8x3 time points = 24), Group B (Dexamethasone treatment; n=8 x 3 time points =24); Group C (Vehicle or Saline control; n=8x3 time points=24); and Naïve animals (n=5). Total n=77. Animals were housed in Madigan Army Medical Center animal facility as per the *Guidelines for the Care and Use of Laboratory Animals of the National Institutes of Health*. Animals obtained were acclimatized for at least 72 hours before use. Vendor health reports indicated that the rats were free of known viral, bacterial and parasitic pathogens [[22](#_ENREF_22)]

All animal experiments were performed according to institutional guidelines with the prior approval by the IACUC. Animals were monitored closely soon after surgery and twice daily thereafter for their health and wellbeing. To relieve pain, animals were injected analgesic buprenorphine (0.03mg/kg) at 12 h intervals for three days post-operatively. We used humane protocol end points in our animal survival study. Animals were sacrificed at pre-determined time points (3, 24 and 120 h) following ischemia-reperfusion procedure. However, if animals showed signs of distress and pain (anorexia, lack of grooming and activity, rapid respiration, a moribund state) not alleviated by analgesics, they were removed from the study and euthanized humanely. The animals were euthanized by injecting Sodium Pentobarbitol (40-80 mg/rat) intraperitoneally.

**9. Housing and Husbandry**

Animals were housed with an inverse 12 hours day-night cycle with lights on at 8:00 pm in a temperature (22±1 ^o^ C) and humidity 55±5%) controlled room. Room temperatures and humidity were checked once a day with 24 hour monitoring system in place. Animals were checked twice a day for evidence of fighting, illness, such as nasal/ocular discharge, diarrhea or lethargy. Proper personnel protective equipment (PPE) was worn by personnel at all times.

Animals were individually housed upon arrival and given an acclimatization period of at least 3 days prior to any procedures. Prior to surgery animals were housed pairwise in type 4 cages filled with hygiene animal bedding and provided enrichment with shepherd shacks, or PVC pipe for climbing [[23](#_ENREF_23)], approved toys or treats, and regular human interaction and gentle handling as daily rounds were performed. All rats were allowed free access to food (maintenance diet) and water in a 12-hour light/dark cycle with room temperature ~ 22 ^o^ C. All cages contained wood shavings, bedding and a tube for environmental enrichment [[24](#_ENREF_24)] . Animals were monitored closely soon after surgery and twice daily thereafter for their health and wellbeing. During the postoperative period, pain was relieved by injecting analgesic buprenorphine (0.03mg/kg) subcutaneously at 12 h intervals for three days.

**10. Sample Size**

Seventy two healthy Wister rats (200-300 g; male) purchased from Harlan Laboratories, Indianapolis, IN, USA were divided into three experimental groups (A, B, C). In each group there were 3 time points (3, 24, 120 h). Group A (VPA treatment; n=8x3 time points = 24), Group B (Dexamethasone treatment; n=8 x 3 time points =24); Group C (Vehicle or Saline control; n=8x3 time points=24); and Naïve animals (n=5). Total n=77.

The sample size used (n=8/group) was similar to previous studies in similar models [[11](#_ENREF_11)]. Also, with an expected variation of approximately 20% among animals in the response (histological kidney damage recovery of grade 0.5 on a scale of 0 to 4) to drug treatment between groups to achieve a power =0.8 to and alpha =0.05 to detect the difference we would require a total of 77 animals [[25](#_ENREF_25)] . Within the study, the experiments were replicated (5 to 8 times) and data were pooled, and compared.

**11. Allocating animals to experimental groups**

Seventy two healthy Wister rats (200-300 g; male) purchased from Harlan Laboratories, Indianapolis, IN, USA were divided into three experimental groups (A, B, C). In each group there were 3 time points (3, 24, 120 h). Group A (VPA treatment; n=8x3 time points = 24), Group B (Dexamethasone treatment; n=8 x 3 time points =24); Group C (Vehicle or Saline control; n=8x3 time points=24); and Naïve animals (n=5). Total n=77.

Animals were randomly assigned to each treatment group starting with group A, B and C. All 3 hour studies were performed first, followed by 24h and 120 h studies. For naïve animal study (control) animals from the pool were randomly picked and euthanized humanely to collect tissue samples for the study. Post-surgical treatments were performed in same order as the surgeries were done to maintain equal time difference between groups in all animals.

**12. Experimental outcomes**

Two primary outcome measures were analyzed: Levels of Creatinine and Blood Urea Nitrogen in the blood plasma; Albumin, Lipocalin 2, Kidney Injury Molecule-1, and Osteopontin in the urine in response to treatment (VPA, Dexamethasone) and no treatment (vehicle or control). Two secondary outcome measures were analyzed: Histologic tissue injury scores in the kidneys with or without treatment; and DNA-microarray analysis of kidney tissues to identify novel gene upregulation or down regulation with or without treatment.

**13. Statistical Methods**

**Kidney Functional Biomarker and Histopathologic Injury Data Analysis:** The data were analyzed for statistical significance and descriptive statistics using *PASW Statistics version 18* software program. The mean values between two were compared by *t* test (Histopathologic score) and among ≥ three groups by One-Way ANOVA with post-hoc (Bonferroni or LSD [Fisher’s Least Square Difference]) correction groups (Creatinine, BUN, Albumin, Lipocalin-2, KIM-1, Osteopontin). All *P*-values presented <0.05 are considered statistically significant, and are two-tailed values.

**Gene Array Data Analysis:** Kidney DNA microarray data were imported into Partek, and Two-Way ANOVA with contrasts at 3, 24 and 120 h was conducted as described previously [[26](#_ENREF_26)]. Raw CEL files were normalized using iterPLIER in Expression Console, which discarded feature sets that performed poorly, similar to the previous report [[27](#_ENREF_27)]. The resulting CHP files were imported into Partek^®^ Discovery Suite^TM^ (Partek, Inc., St. Louis, MO). Affymetrix library files included all available reference files related to RaGene-1_1-st-v1.na33.2.rn4.probeset.csv. A variance-stabilizing transformation (PLIER+16) was applied to the raw intensity (Guide to Probe Logarithmic Intensity Error [PLIER] Estimation) to resolve signal variance following the PLIER analysis. The standard deviation of each transcript ID was determined, the lower 50% were removed, and the remaining 50% of transcript IDs were used as background for pathway analysis. The low variance criteria from Bourgon et al. [[28](#_ENREF_28)] was implemented by computing and sorting the expression variance of each gene over the complete condition set, then removing the low-variance genes (lower half). To determine differentially expressed genes (DEGs), a Three-Way ANOVA was conducted and gene lists were generated by applying a two-fold change cut off and *P*-value with Benjamini-Hochberg False Discovery Rate (FDR) multiple testing (<0.05) correction [[29](#_ENREF_29),[30](#_ENREF_30)]. When few DEGs were identified, DEGs with uncorrected *P*-values were determined for the purpose of pathway analysis.

**Gene Enrichment Analysis:** Gene lists were examined with the Database of Annotation, Visualization and Integrated Discovery (DAVID) [[31](#_ENREF_31)]. Enriched KEGG (Kyoto Encyclopedia of Genes and Genomes) pathways and functional annotation clusters (FACs) were determined using default settings. The most significant biological processes, molecular functions and cellular components from functionally associated clusters with an enrichment score >2.0 were represented and examined. Gene lists were consolidated and sub-categorized to account for redundant annotation terms comprising FACs. For representation purposes, unique transcripts were consolidated from similar FACs.

**14. Baseline Data**

The animals’ health was monitored throughout the experiments by institutional animal health surveillance program according to Animal Welfare Act and Guide for the care and use of laboratory animals [[32](#_ENREF_32)]. The rats were free of all viral, bacterial, and parasitic pathogens listed in the guide for the care and use of laboratory Animals, National Research Council, USA.

**15. Numbers Analyzed**

None of the rats were suspected for any bacterial or viral infections based on clinical health conditions. So no animal was subjected to screening for any infection. All animals used were healthy throughout the study.

**16. Outcomes and Estimation:**  In accordance with the ARRIVE guidelines[[33](#_ENREF_33)], we have reported measures of precision, confidence, and n to provide an indication of significance. Cages of rats were randomly assigned to groups A, B, and C (n=24 per group). The data presented in the report were statistically analyzed. E.g. Histopathologic scores are presented in Table 3.

**Table 3. Histopathologic scores (Mean± SD) of renal cortex and outer medulla at 3, 24 and 120 hours (h) post ischemia-reperfusion (IR) in Valproic Acid (VPA) and Dexamethasone (Dex) treated animals.**

| Group |  | Ischemic Changes  at 3h post-IR | |  | Tubular Necrosis  at 24h post-IR | |  | Tubular Regeneration  at 120h post-IR | |
| --- | --- | --- | --- | --- | --- | --- | --- | --- | --- |
|  |  | **Cortex** | **Medulla** |  | **Cortex** | **Medulla** |  | **Cortex** | **Medulla** |
| Vehicle  (Control) |  | 2.87± | 2.12± |  | 1.75± | 3.12± |  | 2.25± | 2.87± |
|  |  | 1.12^a,1^ | 0.83^a,2^ |  | 1.03^a,1^ | 0.64^a,2^ |  | 1.39^a,1^ | 1.46^a,2^ |
|  |  |  |  |  |  |  |  |  |  |
| VPA |  | 1.57± | 0.86± |  | 1.62± | 2.25± |  | 1.75± | 1.75± |
|  |  | 1.51^b,1^ | 0.9^b,1^ |  | 0.74^a,1^ | 1.28^a,1^ |  | 2.06^a,1^ | 2.06^a,1^ |
|  |  |  |  |  |  |  |  |  |  |
| Dex |  | 2.75± | 1.87± |  | 1.5± | 2.62± |  | 3.43± | 3.71± |
|  |  | 1.03^a,b, 1^ | 0.83^a,2^ |  | 0.92^a,1^ | 0.92^a,2^ |  | 0.53^a,1^ | 0.49^b,1^ |

Mean values within columns among treatment groups with at least one common ***superscript letter (a or b)*** did not vary significantly (P>0.05). Also, mean values in rows between cortex and medulla (within 3h, 24h or 120h post-IR) groups with a common ***superscript*** ***number*** did not vary significantly (P>0.05). Vehicle (saline) served as a control; n=8/sub-group (3, 24 or 120 h).

**17. Adverse events.**

There were no adverse events observed in any of the experimental animals. All animals were euthanized as per the pre-determined time points.

**18. Interpretation/Scientific implications**

In the present study, we analyzed certain significant blood and urine bio-markers of kidney injury, and determined the possible role and mechanisms of VPA and Dex therapy in renal protection. Also, we identified novel genes and signaling pathways involved in kidney IR injury and repair with and without VPA or Dex treatment by DNA microarray analysis.

Plasma biomarkers (creatinine and BUN), and urine bio-markers (albumin, lipocalin-2, KIM-1, and osteopontin) were evaluated with the objective to identify potential early and sensitive markers for the diagnosis of renal damage. Histopathology served as the gold standard for comparison. Blood biomarkers creatinine and BUN were sensitive to detect renal IR injury most definitively by 24 h, which is in agreement with previous reports [[34](#_ENREF_34),[35](#_ENREF_35)].

Reduced histopathologic injury in the renal cortex and medulla at 3 h post-IR confirmed the protective effects of VPA against kidney IR injury. These findings concur with previous reports on kidney IR causing acute kidney injury [[36](#_ENREF_36)] and intestinal IR causing acute lung injury [[37](#_ENREF_37)]. In the present study, at 24 h post-IR, tubular necrosis was significantly pronounced in the medulla than in the cortex in Vehicle controls. However, VPA administration reduced tubular necrosis markedly in the medulla compared to Vehicle control. Interestingly tubular regeneration was lower in VPA group and higher in Dex group compared to Vehicle controls at 120 h post-IR. To our knowledge, other kidney IR studies have not evaluated the differences in tubular necrosis and regeneration between cortical and medullary tubules to compare our results.

Significantly lower levels of pro-inflammatory cytokines IL1β and IL6 in the kidneys of VPA treated animals compared to Vehicle observed in this study is in agreement with other IR injury models [[37-39](#_ENREF_37)]. Increased BCL2 levels at 3 h and /or 24 h post-IR in VPA and Dex treated animals, indicated probable anti-apoptotic mechanism of action in reducing IR injury and these findings are similar to previous reports [[11](#_ENREF_11),[36](#_ENREF_36),[40](#_ENREF_40)]. Additionally, the reduction in inflammatory cytokines in conjunction with the decreased histopathologic IR changes further demonstrates the protective effects of VPA and Dex against kidney IR injury.

To identify novel biomarkers for IR mediated kidney injury and repair, we performed DNA microarray analysis. Stress induced genes (*Hspa1b* and *Hspb1*) and major regulators of apoptosis (*Atf3, Hmox1, and Zfand2a*) were upregulated as early as 3 h following IR injury in all groups (VPA, Dex, and Vehicle). *Havrc1* (KIM-1), a well-known early biomarker of kidney injury was expressed significantly at 24 h post-IR in all groups. *Timp1*, a well-known biomarker of organ injury, expression was also upregulated at 24 h post-IR which is in agreement with the previous report [[36](#_ENREF_36)]. In the present study, high levels of *Havcr1* and *Timp1* expression in Vehicle controls even at 120 h post-IR but not in VPA suggested persistent kidney injury without VPA treatment. Gene expressions observed agree with previously reported studies in many models of kidney injury [[12](#_ENREF_12),[41](#_ENREF_41),[42](#_ENREF_42)]. In animals treated with VPA, stress and apoptosis related gene expressions were far less relative to animals that were untreated (Vehicle controls) or treated with Dex. Our present findings in rat kidney ischemia-reperfusion model (by vascular clamping) further support the previously established roles of VPA in preventing inflammation and apoptosis in ischemia-reperfusion injury [[8](#_ENREF_8),[9](#_ENREF_9),[36](#_ENREF_36),[43](#_ENREF_43)].

Overall, our study provided strong evidence and support for the use of early biomarkers of kidney injury (KIM-1, lipocalin-2, albumin) in the diagnosis of kidney injury. The VPA therapy seems to be promising to mitigate kidney IR injury, essentially indistinguishable from control animals at 120 h. Furthermore, we have presented a comprehensive analysis of microarray data on VPA and Dex induced gene expression in the rat ischemic kidney injury model for the first time.

The model used in our study (45 minutes of ischemia and unilateral nephrectomy) provided high survival rates (≥5 days), thus this resulted in the use of fewer animals compared to more severe models of ischemia-reperfusion injury.

**19. Generalisability/Translation:**  The role of VPA in reducing ischemia-reperfusion injury was demonstrated in this study using a rodent model. We believe VPA is an effective pre-medication in all surgical procedures that involves ischemia/reperfusion of the tissues. However, the findings of this study will serve as a foundation for future studies in large animal models prior to its testing or application in humans. We believe VPA has high translational potential as a therapeutic drug (pre-medication) in humans to prevent ischemia-reperfusion injury as related to surgical procedures.

**20. Funding:** The research performed leading to these results received our institutional funding (Madigan Army Medical Center, Department of the US Army).

**REFERENCES**

1. Sear JW (2005) Kidney dysfunction in the postoperative period. Br J Anaesth 95: 20-32.

2. Coresh J, Selvin E, Stevens LA, Manzi J, Kusek JW, et al. (2007) Prevalence of chronic kidney disease in the United States. JAMA 298: 2038-2047.

3. AmericanCancerSociety (2013) Kidney Cancer: Renal Cell Carcinoma <http://www.cancer.org/cancer/kidneycancer/detailedguide/kidney-cancer-adult-key-statistics>.

4. Howlader N NA, Krapcho M, Garshell J, Miller D, Alterkruse SF, Kosary CL, Yu M, Ruhl J, Tatalovich Z, Mariotto A, Lewis DR, Chen HS, Feuer EJ, Cronin KA (eds). SEER Cancer Statistics Review, 1975-2011, National Cancer Institute, Bethesda, MD.

5. Van Poppel H (2010) Efficacy and safety of nephron-sparing surgery. Int J Urol 17: 314-326.

6. Uzzo RG, Novick AC (2001) Nephron sparing surgery for renal tumors: indications, techniques and outcomes. J Urol 166: 6-18.

7. Zacharias M, Conlon NP, Herbison GP, Sivalingam P, Walker RJ, et al. (2008) Interventions for protecting renal function in the perioperative period. Cochrane Database Syst Rev: CD003590.

8. Kim HJ, Rowe M, Ren M, Hong JS, Chen PS, et al. (2007) Histone deacetylase inhibitors exhibit anti-inflammatory and neuroprotective effects in a rat permanent ischemic model of stroke: multiple mechanisms of action. J Pharmacol Exp Ther 321: 892-901.

9. Butt MU, Sailhamer EA, Li Y, Liu B, Shuja F, et al. (2009) Pharmacologic resuscitation: cell protective mechanisms of histone deacetylase inhibition in lethal hemorrhagic shock. J Surg Res 156: 290-296.

10. Zacharias N, Sailhamer EA, Li Y, Liu B, Butt MU, et al. (2011) Histone deacetylase inhibitors prevent apoptosis following lethal hemorrhagic shock in rodent kidney cells. Resuscitation 82: 105-109.

11. Kumar S, Allen DA, Kieswich JE, Patel NS, Harwood S, et al. (2009) Dexamethasone ameliorates renal ischemia-reperfusion injury. J Am Soc Nephrol 20: 2412-2425.

12. Charlton JR, Portilla D, Okusa MD (2014) A basic science view of acute kidney injury biomarkers. Nephrol Dial Transplant.

13. Schiffl H, Lang SM (2012) Update on biomarkers of acute kidney injury: moving closer to clinical impact? Mol Diagn Ther 16: 199-207.

14. Vaidya VS, Ferguson MA, Bonventre JV (2008) Biomarkers of acute kidney injury. Annu Rev Pharmacol Toxicol 48: 463-493.

15. Mishra J, Ma Q, Prada A, Mitsnefes M, Zahedi K, et al. (2003) Identification of neutrophil gelatinase-associated lipocalin as a novel early urinary biomarker for ischemic renal injury. J Am Soc Nephrol 14: 2534-2543.

16. Persy VP, Verstrepen WA, Ysebaert DK, De Greef KE, De Broe ME (1999) Differences in osteopontin up-regulation between proximal and distal tubules after renal ischemia/reperfusion. Kidney Int 56: 601-611.

17. Zhang PL, Rothblum LI, Han WK, Blasick TM, Potdar S, et al. (2008) Kidney injury molecule-1 expression in transplant biopsies is a sensitive measure of cell injury. Kidney Int 73: 608-614.

18. Schena M, Shalon D, Davis RW, Brown PO (1995) Quantitative monitoring of gene expression patterns with a complementary DNA microarray. Science 270: 467-470.

19. Godet C, Goujon JM, Petit I, Lecron JC, Hauet T, et al. (2006) Endotoxin tolerance enhances interleukin-10 renal expression and decreases ischemia-reperfusion renal injury in rats. Shock 25: 384-388.

20. Delbridge MS, Shrestha BM, Raftery AT, El Nahas AM, Haylor JL (2007) Reduction of ischemia-reperfusion injury in the rat kidney by FTY720, a synthetic derivative of sphingosine. Transplantation 84: 187-195.

21. Fukudome EY, Li Y, Kochanek AR, Lu J, Smith EJ, et al. (2012) Pharmacologic resuscitation decreases circulating cytokine-induced neutrophil chemoattractant-1 levels and attenuates hemorrhage-induced acute lung injury. Surgery 152: 254-261.

22. Katayama M, Jiamsripong P, Bukatina AE, Lombari TR, McMahon EM, et al. (2013) Optimized administration regimen of lopinavir for a myocardial ischaemia reperfusion study in Sprague-Dawley rats. Lab Anim 47: 122-126.

23. Jung F, Stephan KE, Backes H, Moran R, Gramer M, et al. (2013) Mismatch responses in the awake rat: evidence from epidural recordings of auditory cortical fields. PLoS One 8: e63203.

24. Meakin LB, Sugiyama T, Galea GL, Browne WJ, Lanyon LE, et al. (2013) Male mice housed in groups engage in frequent fighting and show a lower response to additional bone loading than females or individually housed males that do not fight. Bone 54: 113-117.

25. Sena ES, Jeffreys AL, Cox SF, Sastra SA, Churilov L, et al. (2013) The benefit of hypothermia in experimental ischemic stroke is not affected by pethidine. Int J Stroke 8: 180-185.

26. Ha K, Coulombe-Huntington J, Majewski J (2009) Comparison of Affymetrix Gene Array with the Exon Array shows potential application for detection of transcript isoform variation. BMC Genomics 10: 519.

27. Qu Y, He F, Chen Y (2010) Different effects of the probe summarization algorithms PLIER and RMA on high-level analysis of Affymetrix exon arrays. BMC Bioinformatics 11: 211.

28. Bourgon R, Gentleman R, Huber W (2010) Independent filtering increases detection power for high-throughput experiments. Proc Natl Acad Sci U S A 107: 9546-9551.

29. Hochberg Y, Benjamini Y (1990) More powerful procedures for multiple significance testing. Stat Med 9: 811-818.

30. Reiner A, Yekutieli D, Benjamini Y (2003) Identifying differentially expressed genes using false discovery rate controlling procedures. Bioinformatics 19: 368-375.

31. Dennis G, Jr., Sherman BT, Hosack DA, Yang J, Gao W, et al. (2003) DAVID: Database for Annotation, Visualization, and Integrated Discovery. Genome Biol 4: P3.

32. ILAR (2011) Guide for the Cae and Use of Laboratory Animals. National Research Council Eighth Edition.

33. Kilkenny C, Browne W, Cuthill IC, Emerson M, Altman DG, et al. (2010) Animal research: reporting in vivo experiments: the ARRIVE guidelines. Br J Pharmacol 160: 1577-1579.

34. Basireddy M, Isbell TS, Teng X, Patel RP, Agarwal A (2006) Effects of sodium nitrite on ischemia-reperfusion injury in the rat kidney. Am J Physiol Renal Physiol 290: F779-786.

35. Chatterjee PK, Brown PA, Cuzzocrea S, Zacharowski K, Stewart KN, et al. (2001) Calpain inhibitor-1 reduces renal ischemia/reperfusion injury in the rat. Kidney Int 59: 2073-2083.

36. Van Beneden K, Geers C, Pauwels M, Mannaerts I, Verbeelen D, et al. (2011) Valproic acid attenuates proteinuria and kidney injury. J Am Soc Nephrol 22: 1863-1875.

37. Kim K, Li Y, Jin G, Chong W, Liu B, et al. (2012) Effect of valproic acid on acute lung injury in a rodent model of intestinal ischemia reperfusion. Resuscitation 83: 243-248.

38. Liu Z, Li Y, Liu B, Deperalta DK, Zhao T, et al. (2013) Synergistic effects of hypertonic saline and valproic acid in a lethal rat two-hit model. J Trauma Acute Care Surg 74: 991-997; discussion 997-998.

39. Liu Z, Li Y, Chong W, Deperalta DK, Duan X, et al. (2014) Creating a prosurvival phenotype through a histone deacetylase inhibitor in a lethal two-hit model. Shock 41: 104-108.

40. Zhang Z, Qin X, Zhao X, Tong N, Gong Y, et al. (2012) Valproic acid regulates antioxidant enzymes and prevents ischemia/reperfusion injury in the rat retina. Curr Eye Res 37: 429-437.

41. Vlasakova K, Erdos Z, Troth SP, McNulty K, Chapeau-Campredon V, et al. (2014) Evaluation of the relative performance of 12 urinary biomarkers for renal safety across 22 rat sensitivity and specificity studies. Toxicol Sci 138: 3-20.

42. Vaidya VS, Ozer JS, Dieterle F, Collings FB, Ramirez V, et al. (2010) Kidney injury molecule-1 outperforms traditional biomarkers of kidney injury in preclinical biomarker qualification studies. Nat Biotechnol 28: 478-485.

43. Causey MW, Salgar S, Singh N, Martin M, Stallings JD (2012) Valproic acid reversed pathologic endothelial cell gene expression profile associated with ischemia-reperfusion injury in a swine hemorrhagic shock model. J Vasc Surg 55: 1096-1103 e1051.
